# Structural Determinants of Peptide Nanopore Formation

**DOI:** 10.1021/acsnano.4c02824

**Published:** 2024-06-06

**Authors:** Leisheng Sun, Kalina Hristova, Ana-Nicoleta Bondar, William C. Wimley

**Affiliations:** † Department of Biochemistry and Molecular Biology, Tulane University School of Medicine, New Orleans, Louisiana 70112, United States; ● Department of Materials Science and Engineering, Whiting School of Engineering, Johns Hopkins University, Baltimore, Maryland 21218, United States; ∥ Institute for NanoBioTechnology, Johns Hopkins University, Baltimore, Maryland 21218, United States; ⊥ Faculty of Physics, University of Bucharest, Atomiştilor 405, Măgurele 077125, Romania; # Forschungszentrum Jülich, Institute of Computational Biomedicine, IAS-5/INM-9, Wilhelm-Johnen Straße, 5428 Jülich, Germany

**Keywords:** Nanopore, pore-forming, peptide, membrane, selectivity, molecular dynamics, hydrogen bonding

## Abstract

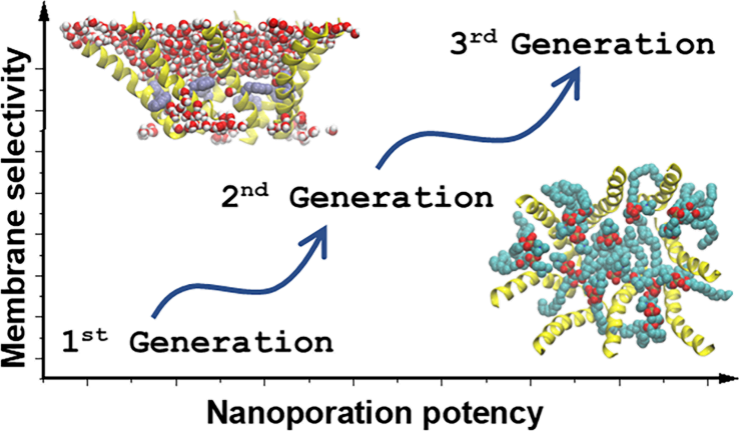

We have evolved the nanopore-forming macrolittin peptides from the bee venom peptide melittin using successive generations of synthetic molecular evolution. Despite their sequence similarity to the broadly membrane permeabilizing cytolytic melittin, the macrolittins have potent membrane selectivity. They form nanopores in synthetic bilayers made from 1-palmitoyl, 2-oleoyl-phosphatidylcholine (POPC) at extremely low peptide concentrations and yet have essentially no cytolytic activity against any cell membrane, even at high concentration. Here, we explore the structural determinants of macrolittin nanopore stability in POPC bilayers using atomistic molecular dynamics simulations and experiments on macrolittins and single-site variants. Simulations of macrolittin nanopores in POPC bilayers show that they are stabilized by an extensive, cooperative hydrogen bond network comprised of the many charged and polar side chains interacting with each other via bridges of water molecules and lipid headgroups. Lipid molecules with unusual conformations participate in the H-bond network and are an integral part of the nanopore structure. To explore the role of this H-bond network on membrane selectivity, we swapped three critical polar residues with the nonpolar residues found in melittin. All variants have potency, membrane selectivity, and cytotoxicity that were intermediate between a cytotoxic melittin variant called MelP5 and the macrolittins. Simulations showed that the variants had less organized H-bond networks of waters and lipids with unusual structures. The membrane-spanning, cooperative H-bond network is a critical determinant of macrolittin nanopore stability and membrane selectivity. The results described here will help guide the future design and optimization of peptide nanopore-based applications.

## Introduction

The 26-residue peptide melittin is the most abundant compound by weight in the venom of the Western Honeybee (*Apis mellifera*) which has the most cytotoxic venom of any characterized bee/wasp species.^1^ Melittin is a membrane-lytic toxin that presumably evolved to cause pain and tissue destruction in any intruder that threatens a beehive, including insects, reptiles, amphibians, birds, and mammals. Melittin folds into an amphipathic α-helical secondary structure when bound to lipid membranes. The strong partitioning of the amphipathic helix into membranes disrupts lipid packing, resulting in nonspecific membrane permeabilization without the formation of explicit transmembrane pores.^2^ Melittin has been extensively studied because it has a wide variety of potentially useful bioactivities.^1^ To increase the usefulness of melittin or the selectivity of melittin activity against different cells, such as cancer cells, researchers have tested many approaches,^1,3,4^ including using subtoxic concentrations, caging, conjugating to specific carrier molecules, or formulation in gels, liposomes, nanoparticles, or other structures that improve its effects or reduce its toxicity.

We have been using synthetic molecular evolution, high-throughput screening of iterative peptide libraries, to discover gain-of-function of analogues of melittin that broaden the potential utility of melittin-derived, membrane-permeabilizing peptides.^5−8^ The first-generation rational combinatorial library containing 7,776 members was based directly on the sequence of melittin. We screened this library for melittin variants that form *equilibrium* pores in synthetic 1-palmitoyl, 2-oleoyl-phosphatidylcholine (POPC) liposomes at low concentrations where melittin is not active. By this approach we identified a set of highly active melittin analogues.^5,9^ The most active of these analogues, called Melp5, differs from the parent melittin in only 5 of its 26 residues, most of which make MelP5 more ideally amphipathic. Like melittin, MelP5 has potent permeabilizing activity against synthetic vesicles of many different lipid compositions and is highly cytolytic against bacterial and mammalian cells.^10,11^ Importantly for the next generation of peptides, MelP5 was shown to release macromolecules from lipid vesicles,^9^ a property with significant utility in biotechnology.

Subsequently, a second-generation library of 18,432 members was created using MelP5 as a template. In this generation, acidic amino acids were possible in six sites with *i* to *i* + 3 and *i* to *i* + 4 helical spacings, which placed them on a polar face of the α-helix of MelP5. We screened this library in two separate ways for the most potent macromolecule release activity, or nanoporation. First, we screened for pH-triggered macromolecular poration which occurred at pH 5 in peptides that were inactive at pH 7. By this approach we discovered the pHD peptides^7,12,13^ which have 5 or 6 of the 6 possible acidic residues. Second, we screened for very potent macromolecular poration at neutral pH 7, identifying the macrolittins^8,10^ which form nanopores and release macromolecules without acidification. The macrolittins all contain 3 of the 6 possible acidic amino acids. They induce macromolecule-sized nanopores in lipid bilayers at a strikingly low peptide-to-lipid ratio (P:L) ≤ 1:1000. A major difference between melittin and these two generations of variants is that the variants assemble into membrane-spanning helical structures, while melittin generally does not.^5,7^ Melittin, MelP5, and the macrolittins all fold into amphipathic α-helices in membranes, but macrolittin structure and function are dominated by the presence of a continuous, highly polar surface containing 3 charged acidic residues, 2 basic residues, and 4 or 5 other polar residues, including the highly polar glutamine. Some of the these charged/polar residues are highly conserved in the high-throughput screens that gave rise to the pHD peptides and the macrolittins.

Macrolittins are highly membrane selective, with the most potent activity in POPC, the bilayers against which they were evolved.^10^ Unlike MelP5, macrolittins are much less capable of permeabilizing synthetic membranes containing cholesterol or bilayers that are even slightly thicker than POPC. Similarly, while melittin and MelP5 are highly cytotoxic, macrolittins have no measurable lytic activity against mammalian cells even at high peptide concentration, despite their very high potency against synthetic POPC bilayers.^10^ This dramatic 25,000-fold^10^ increase in selectivity for synthetic POPC bilayers over cell plasma membranes^10^ suggests that macrolittin nanopores could be useful in many biotechnological areas such as triggered-release of cargo from liposomes *in vivo*.^10^

In this work, we investigate how the unusual macrolittin nanopore structure is stabilized by the charged and polar residues in POPC and how this structure leads to the extreme membrane selectivity of the macrolittins. We use atomistic molecular dynamics (MD) simulations in POPC and experiments in various bilayers and cells to study the unusual structural determinants of the macrolittin nanopores. Guided by the simulations and the sequence conservation among the pHD peptides and macrolittins, we experimentally compare the activities of MelP5, two macrolittins called M70 and M159, and three variants in which we individually swap amino acids that are consistently changed from nonpolar to polar/charged during the evolution of macrolittins (and pHD peptides) from MelP5. These residues appear in simulations to be critical nodes in the extensive membrane-spanning hydrogen (H)-bond network of the nanopores. Experiments show that all three varied polar residues contribute significantly to the stability and membrane selectivity of the macrolittin nanopores. Based on these simulations and experiments, we propose a model in which the macrolittin nanopore structure is stabilized by an extensive, cooperative network of H-bonds between peptide side chains, water, and lipid molecules that spans the entire membrane thickness. This structural model provides insights that will help enable the design and screening of even more useful peptide nanopores.

## Results and Discussion

### Sequence Features of the Macrolittins

**Figure 1:**
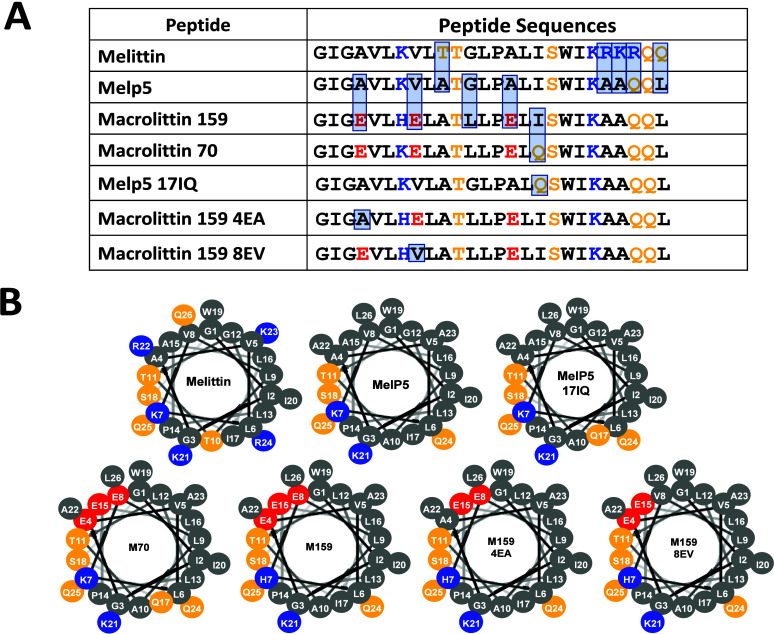
Bee venom toxin melittin and its variants. (A) List of the amino acid sequences and net charges of main toxin melittin and variants studied here. Acidic (red), basic (blue), and polar (yellow) residues are shown to highlight amphipathicity. (B) Helical wheel projections of the peptides discussed in this work. Helical wheels are idealized representations of the side-chain positions, assuming that all residues are canonically α-helical. Deviations from these idealized geometries are likely, especially due to the conformational effects of the proline residue at position 14, and from the potential nonhelical structure of residues on the helix ends.

To explain the loss of cell toxicity in the evolution of the macrolittins from melittin and MelP5, we first compared the sequences of the three generations (Figure 1A). Between melittin and MelP5 there are five amino acid changes which make MelP5 more ideally amphipathic than melittin (Figure 1A), but they are otherwise very similar in physical chemical properties. The critical changes include extension of the amphipathic helix to the C-terminus by the K23A change and the narrowing of the polar face by the T10A change.^5^ For reference the position of these polar groups are shown on idealized helical wheel diagrams in Figure 1B, although we note that the helix-breaking proline at position 14 could change the alignment of the N- and C-terminal helices.

In the second generation of synthetic molecular evolution, MelP5 was used as the template for a library from which both the pHD peptides and macrolittins were selected. In this library, residues 4, 8, 11, 12, 15, and 18 had the possibility of being acidic aspartate (D), glutamate (E), or the native residue in MelP5. This library also had the possibility of having glutamine (Q) at position 17 instead of the native isoleucine (I), as well as other changes. From this library 10 pHD peptides and 5 macrolittins were selected in two parallel screens, as described eslewhere.^7,8^ The two families have nearly identical nanoporation activities, except that the pHD peptides, with 5 acidic residues, become active at acidic pH ≤ 5.5, while the macrolittins, with 3 acidic residues, are active at pH 7. Every selected peptide from either family, with one exception, had acidic residues at both positions 4 and 8 instead of the native nonpolar alanine and valine residues in MelP5. The number of additional acidic residues selected was constant, but their positions were variable. The acidic residues at positions 4 and 8 must support nanoporation activity in both families of peptides. Further highlighting the importance of these residues, 20/29 (70%) of the selected acidic residues in positions 4 and 8 are glutamate, showing a strong preference for the acidic side chain that is one methylene unit longer. Furthermore, 14/15 selected pHD/macrolittin sequences have Q17 selected instead of the native I17 present in MelP5, another highly statistically significant preference.^7,8^ In this work we study macrolittins M70 and M159, which are very similar. M70 contains E4, E8, and Q17 residues. M159 also has E4 and E8 but is the only macrolittin with isoleucine at position 17. Despite this, M159 has activity that is indistinguishable from the other macrolittins.^8^

### Structure of the Macrolittin Nanopore from MD Simulations

To probe the structure of the macrolittin peptide nanopores and determine how the many acidic and polar residues might support nanoporation activity and membrane selectivity, we performed all-atom MD simulations of 8-mer pores of the two macrolittins M70 and M159 in hydrated POPC bilayers and used coordinates from the MD trajectories to compute dynamic H-bond networks with a graph-based algorithm.^14,15^ MelP5 has been simulated previously.^16^

In the simulations, we initially placed eight peptides in a membrane-spanning configuration in hydrated POPC bilayers. The peptides initially had the same internal coordinates and transmembrane orientations, which were those previously sampled by the closely related pHD15 peptide in a pore-compatible membrane.^12^ According to our estimations, eight peptides placed equidistantly in transmembrane orientations such that they lack direct contacts with each other delineate a periphery whose diameter is compatible with the smaller pHD pores observed experimentally. When generating the initial coordinates of the peptide–membrane–water simulation systems we allowed for lipid molecules, but not water, in the region delineated by the periphery of the pore. In this way we initiated simulations in a hypothetic state of a bilayer with peptides inserted as monomers across the bilayer but prior to any peptide hydration, self-assembly, or nanopore formation in the bilayer, which allows bulk water molecules to enter the region delineated by the peptides. In the context of these initial conditions, nanopore formation requires that the peptides and lipids sample structures with a stable pore periphery.

**Figure 2:**
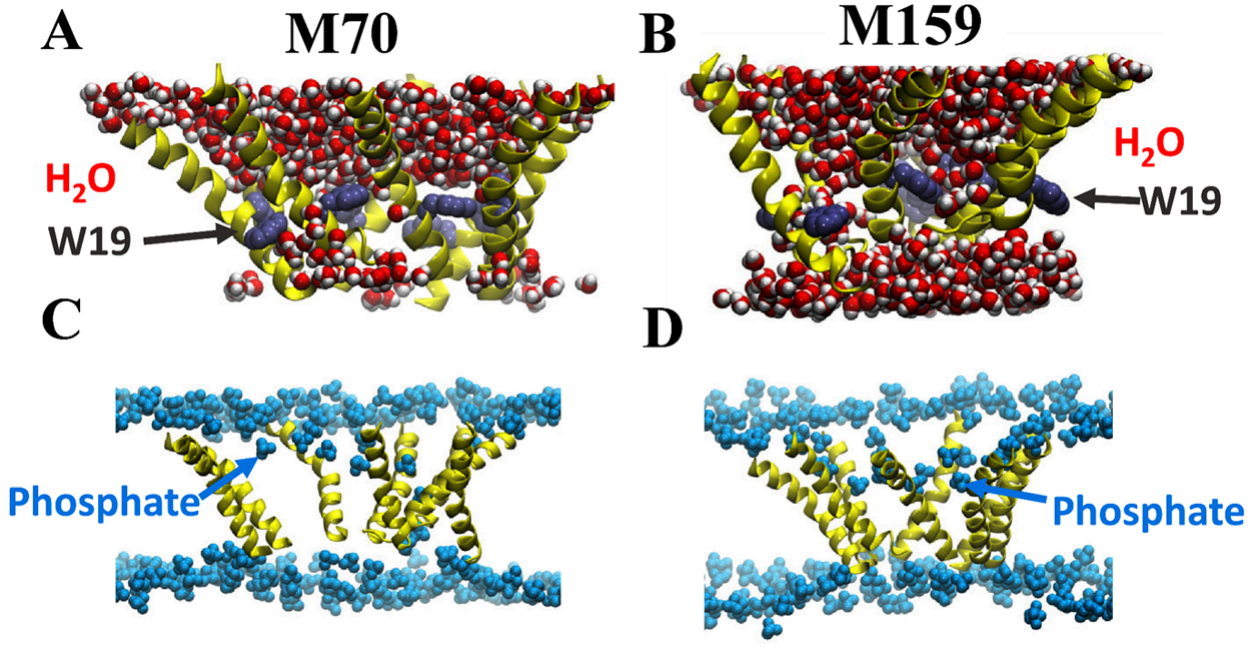
Lipid and water interactions inside macrolittin pores. The structure snapshots are from a 510 ns simulation of M70 (A and C) and a 500 ns simulation M159 (B and D). Peptides are shown in yellow. (A and B) Water molecules near the pore are shown in red and white spacefill. The side chain of residue W19 is shown in gray to indicate the depth of peptide in the bilayer; for clarity, H atoms are not shown. (C and D) Lipid phosphate groups are shown in blue. The left images are for macrolittin M70, while the right images are for M159.

The two macrolittin peptides rapidly evolved into a nanopore-like state during the simulations. Many water molecules move into the membrane plane where, by H-bonding with the polar residues of the peptides and lipid headgroups, they help an extensive network of H-bonds that stabilizes the periphery of the pore. In Figure S1 we show time courses of the number of direct H-bonds between peptide side chains and of the water bridges between the side chains. There are no fewer than about 60–80 such H-bonds in both the M70 and M159 macrolittin simulations (Figure S1); thus, there are about 10 direct and water-mediated side-chain bridges per peptide, which suggests extensive H-bond connectivity within the pore. To this large number of H-bonds we must add those mediated by lipid molecules that are closely associated with the pore. As illustrated by the snapshots of the pore structures after about 0.5 μs (Figure 2), M70 and M159 sustain complex, yet stable pore architectures whereby the peptides delineate a large, central cavity that hosts numerous water molecules and lipid molecules. The polar and charged peptide residues, including the critical polar groups E4, E8, and Q17 (in M70), remain oriented toward the interior of the pore (Figures 2A,B). In both simulations, bulk water has entered the central pore and is connected across the bilayer, but lipid molecules also still occupy the pore. The location of W19, in gray spacefill (Figure 2A,B), provides a visual indication of the insertion of the peptides into the bilayer. For both M70 and M159, W19 residues remain inserted into the bilayer hydrocarbon. As we will see below, in unstable pore formers with mutated E4 or E8 aspartates, some W19 residues become interfacial as peptides move toward probable deinsertion from the bilayer.

Both the M70 and M159 pore structures show the characteristic bend in the middle of the helix caused by the helix-breaking P14 residue. We showed previously that P14 is essential for MelP5 activity,^5^ and others have shown that P14 is essential for the structure^17^ and activity^18^ of melittin. For these reasons, we did not vary P14 in the MelP5-based library that gave rise to the macrolittins.

**Figure 3:**
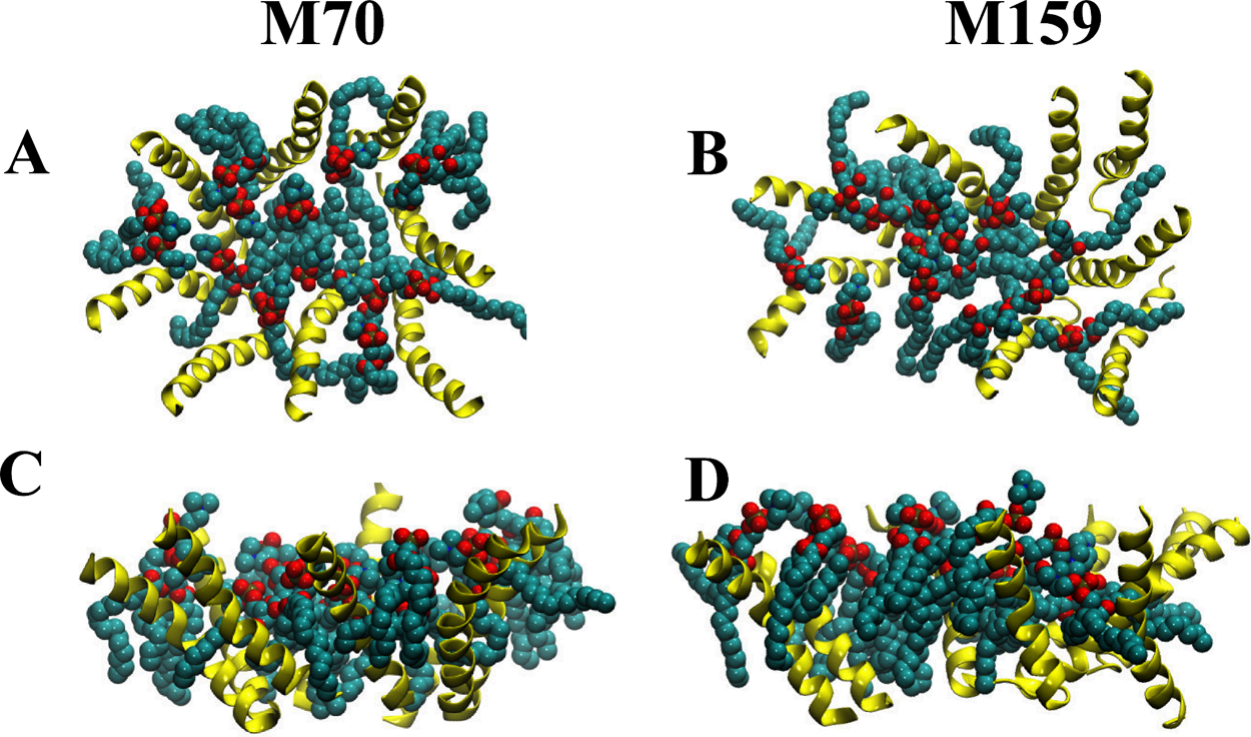
Pore-associated lipids in macrolittin pore structure. These structure snapshots are from the equilibrated structures after 480–500 ns of simulation for M70 and M159. Peptides are shown in yellow. Lipid molecules are shown as van der Waals spheres with carbon atoms colored cyan, and oxygen atoms are red. (A and B) Top down view of M70 and M159 pores showing lipid molecules associated with the pore. (C and D) Side view of M70 and M159 pores showing lipid molecules associated with the pore. Note that, for clarity, only lipids of one leaflet are shown; lipid phosphate groups of both bilayer leaflets are shown in Figure 2C,D.

In Figure 2C,D we show the lipids associated with macrolittin nanopores. Peptide helices are yellow, and lipid phosphate groups are blue. These images show that the macrolittin nanopores contain multiple lipid phosphates located deep in the bilayer plane. These phosphates are from lipids in the pore that have highly unusual conformations and orientations and that intimately participate in the structure of the nanopore. In Figure 3A,B examples of conformations sampled by these unusual lipids are shown in top views, and in Figure 3C,D are shown side views of the M70 and M159 pore structures. Lipid phosphates are shown in red and the acyl chains in cyan. Lipids with phosphates groups deep in the bilayer can even have their acyl chains oriented roughly parallel to the bilayer plane.

### H-Bond Networks Stabilize Peptide Nanopores

The preponderance of charged and polar side chains in the membrane is an unusual feature of the macrolittin peptide nanopores. The macrolittins are distinguished from most membrane-spanning helices by their very high abundance of polar and charged residues, including having three charged acidic residues; two basic residues; as well as multiple polar T, S, and Q residues. All along the polar face of the macrolittins, the polar/charged residues are aligned by virtue of their *i* + 3 and *i* + 4 helical spacings (Figure 1).

**Figure 4:**
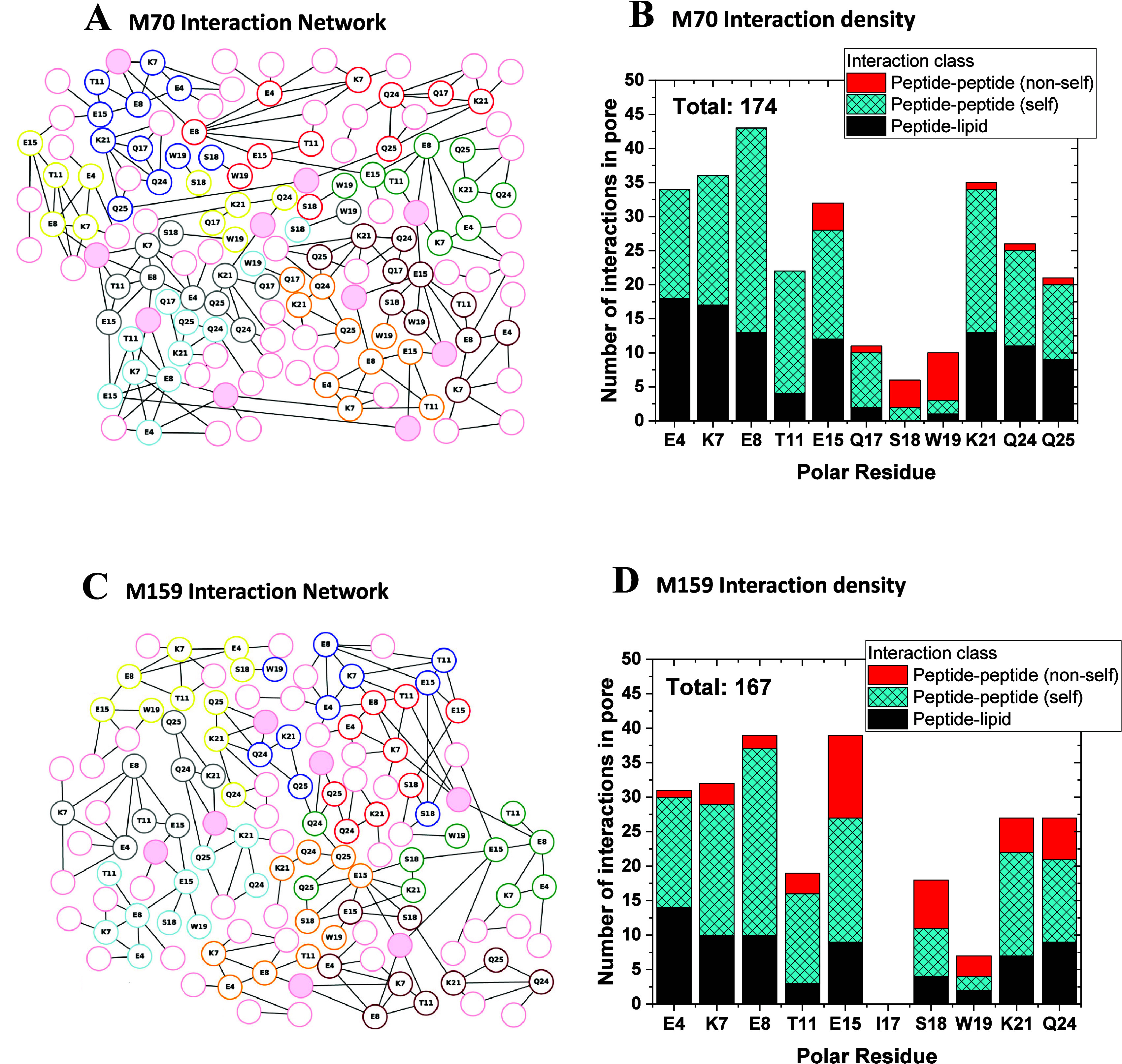
H-bond networks of the M70 and M159 macrolittins. H-bonds with up to three H-bonded water bridges, and with at least 30% occupancy, were counted over the last 200 ns of each 500−510 ns simulation. (A) H-bond graph of macrolittin M70. Nodes representing lipid phosphate groups are shown as pink circles; full pink circles represent lipid phosphate groups that connect to two distinct peptides. Graph nodes colored other than pink represent peptide side chains. Lines (edges) between two graph nodes represent H-bonds, which can be direct or mediated by bridges of up to three H-bonding water molecules. Lipid nodes and edges that lack connections to peptide nodes, and only indicate lipid–lipid interactions, are excluded from the graph. (B) Number of interactions in the M70 pore, presented as the sum of interactions per residue, for all eight peptides of the pore. (C) H-bond graph for the M159 pore. (D) H-bond density for the M159 pore. H-bond graphs corresponding to panels A and C, but with detailed information about H-bond occupancies and average water numbers, are presented in the Supporting Information.

The simulations suggest that the nanopore structure of the macrolittins is stabilized by a cooperative network of H-bond interactions between the abundant polar and charged groups, typically bridged by lipids and water molecules. In Figure 4 we characterize the H-bond networks observed for M70 and M159. We show the abundance of intra- and intermolecular interactions between the polar side chains for M70 (Figure 4A,B) and for M159 (Figure 4C,D). To simplify the display of the H-bond networks, we show only H-bonds that are sampled during at least 30% of the coordinate sets used for analyses. While this 30% occupancy cutoff applied in Figure 4 might appear somewhat arbitrary, it allows for an overall robust agreement between H-bond networks sampled in repeat simulations we initiated from the same starting coordinates as described above, but with different initial velocity assignments. Repeat simulations results, described in detail in Figures S1–S7, produce structures, H-bond networks, H-bond densities, and time courses that agree with the main simulations.

For main and repeat simulations of M70 and M159, we show in Figures S4–S7, the average number of waters that bridge each H-bond and the average % occupancy of those interactions. These images show that most H-bond connections shown in the H-bond graph have water bridges of roughly 2 waters. These plots reveal many interesting details of the nanopore H-bond networks. While the main feature of these networks is the large number of promiscuous interactions, a few interactions are especially stable or well populated. For example, H-bond connections between S18 and W19 residues of distinct peptides are relatively common and are especially stable, typically with ≤1 intervening waters, and can have occupancy values over 50% and as high as >90% (see, e.g., the red-green, cyan-gray, and gray-yellow S18–W19 pairs in the center of the H-bond graph shown in Figure 4A, and the yellow-blue and blue-red S18–W19 pairs in Figure S2E). S18 can also bridge to E15 (see, e.g., the brown nodes in Figures 4A and S2E).

At the N-termini of M70 and M159, E4, K7, E8, and T11 of a peptide tend to participate in H-bond clusters with lipids and water, which can bridge further to a nearby peptide. Likewise, at the C-termini, K21, Q24, and Q25 of one peptide participate in both intra- and interpeptide H-bond clusters with lipids and water molecules, such that some of the neighboring peptides bridge via such K21–Q24–Q25 H-bond clusters (see, e.g., the blue and red nodes in Figures 4A and S3E). In M159, the K21–Q24–Q25 triads of no fewer than 5 peptides interconnect with each other, with lipids and water (see yellow, blue, red, green, and orange nodes in Figure 4C); in the repeat M159 simulation, the K21–Q24–Q25 triads of three peptides interconnect via lipids and water (see the green, brown, and orange nodes in Figure S3E). Taken together, the H-bond graph computations for M70 and M159 indicate an extensive participation of the Glu, Gln, and Lys side chains in interpeptide H-bond networks with water molecules and lipid phosphate groups, suggesting these residues are important structural determinants of pore stability.

### Selection of Residues That Were Varied to Test the Importance of the Complete H-Bond Network

In this work, we opted to test the effects of varying the three most conserved polar residues found during the evolution of the macrolittins and pHD peptides from MelP5. First, we reverted the glutamate residues E4 and E8 of M159 back to the nonpolar residues alanine and valine, respectively, that are found in MelP5 and melittin. We show in Figure 4 that these residues are important nodes in the H-bond network that stabilize the macrolittin nanopore. Because they were consistently selected in the evolution of the macrolittins, we hypothesize that they are critical for the stability of the nanopore and thus for the membrane selectivity of the macrolittins. Second, we changed the I17 of MelP5 into the Q17 found in all but one of the macrolittin and pHD peptides. Q17 is also a node in the H-bond networks that stabilizes the peptide nanopores (Figure 4). Next, we compare the biophysical and cellular activities of all six peptides, M159, M70, MelP5, MelP5 I17Q, M159 E4A, and M159 E8V, and below we simulate their structures in POPC bilayers.

### Synthetic Membrane Permeabilization by Parent Peptides and Variants

**Figure 5:**
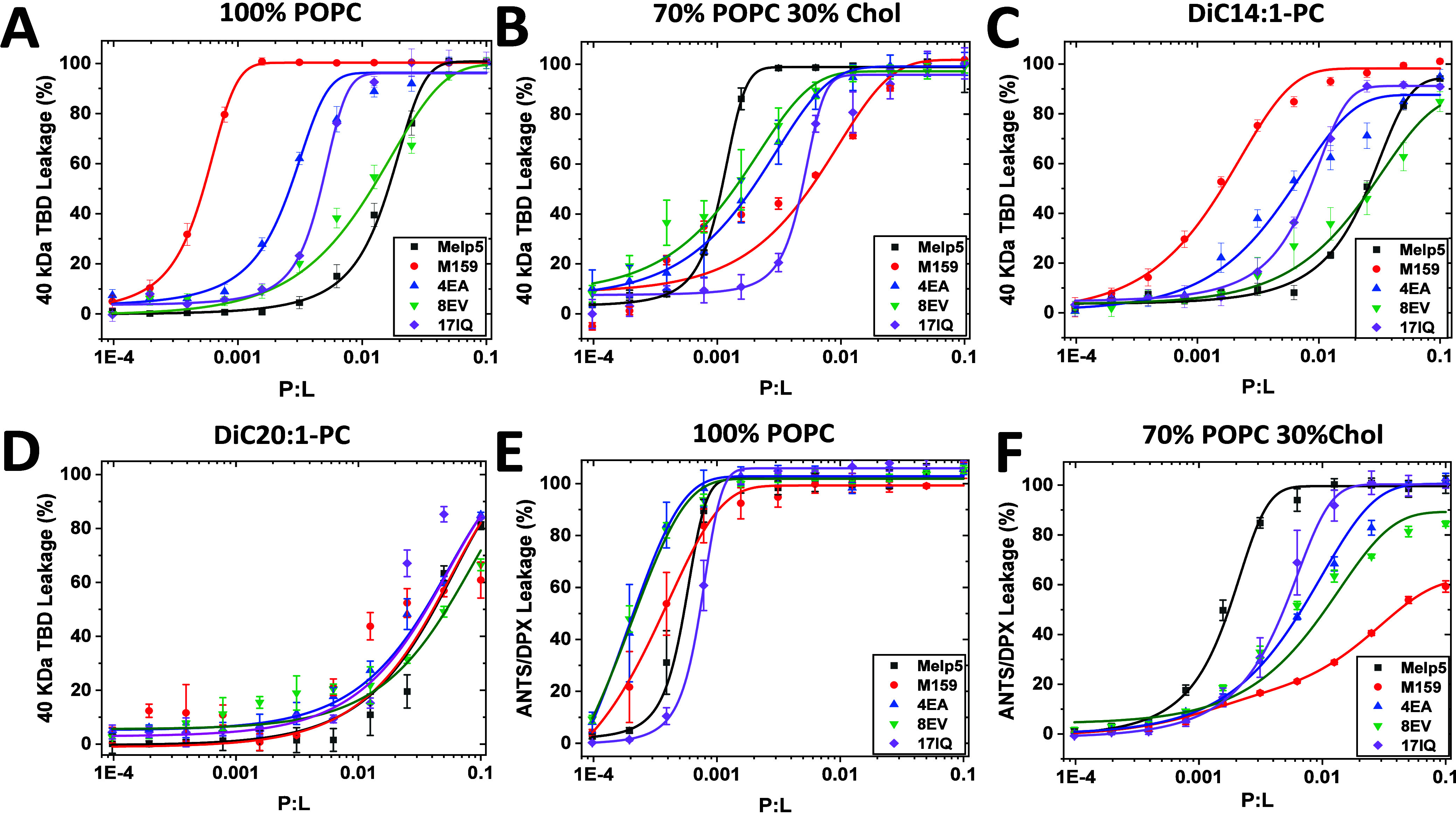
Leakage of different vesicles induced by peptides. (A and B) ANTS/DPX small molecule leakage results. Peptides were incubated with liposomes composed of 100% POPC (A) or 70% POPC and 30% Cholesterol (B). Vesicles contained encapsulated ANTS (fluorophore) and DPX (quencher). Results are measured after 1 h. (C–F) Macromolecular TAMRA-Biotin-Dextran 40 kDa (TBD) leakage assay. Peptides were incubated with different compositions of liposomes: 100% POPC (C); 70% POPC and 30% cholesterol (D); 100% diC14:1-PC (E); 100% diC20:1-PC (F). Vesicles contain entrapped TBD and AlexaFluor488-streptavidin are added to the external solution. Results are shown after 1 h of incubation. In all experiments, Triton X100 was added to obtain the 100% leakage value as a positive control.

Here, we explore the permeabilization of different lipid bilayers by M159, MelP5, and the three variants to test the effects of the E4A and 8EV changes on membrane selectivity. First, we examine leakage of a macromolecule, TAMRA-biotin-dextran (TBD) 40 kDa, as described previously^7−9,12,13^ to assess the nanopore-forming properties of the peptides. Macromolecule leakage at low peptide concentration is a readily observable functional property of the macrolittin peptide nanopores. This is shown in Figure 5A where M159 caused TBD leakage from POPC bilayers at a peptide-to-lipid ratio (P:L) < 0.001. MelP5, on the other hand, causes TBD leakage from POPC only at P:L > 0.01. MelP5 is thus about 30-fold less potent than M159 in this assay. The three variants all have activities in POPC that are intermediate to the parent peptides. MelP5 I17Q is about 7-fold more potent than MelP5 but much less potent than M159. M159 E4A is about 7-fold less potent than M159 but more potent than MelP5. M159 V8E is 25-fold less potent than M159, with activity that is similar to MelP5 in this assay.

Bilayer hydrocarbon core properties affect macrolittin and MelP5 activity in very different ways.^10^ We show this effect in Figure 5B where the membrane selectivity and potency of M159 to release TBD from POPC vesicles is substantially decreased by the addition of 30% cholesterol, while the activity of MelP5 is significantly increased by cholesterol. In POPC bilayers with 30% cholesterol, MelP5 is more active than M159. Despite the swapped relative activities of MelP5 and M159, the three variants again have intermediate TBD release activities in this bilayer system. Like the observations for POPC bilayers, the activity of M159 E4V in POPC+30% cholesterol is more similar to the activity of M159, while M159 8EV has activity that is more similar to that of MelP5.

Against diC14:1PC bilayers, which are fluid phase bilayers that are slightly thinner than POPC^12^ (C16:0,C18:1 PC), M159 retains potent TBD release and nanoporation activity (Figure 5C) while MelP5 has much lower activity. Like the case for POPC bilayers, the variants have intermediate activities. On the other hand, against diC20:1 PC bilayers that are slightly thicker than POPC,^12^ macromolecule leakage activity, and thus nanopore formation, is greatly reduced, occurring only at P:L ≫ 0.01.^10^ In Figure 5D we show the lack of nanoporation in diC20:1 PC bilayers by M159 and MelP5. In this case, the variants also have little activity in this lipid. These results show that the specific properties of POPC bilayers are important for macrolittin nanoporation activity, validating our choice of POPC for the simulations.

In Figure S8 we show that macrolittins and variants bind strongly to all lipid compositions studied, including diC20:1 bilayers in which they have very low nanoporation activity. In Figure S9 we show with circular dichroism spectra that the macrolittins and variants have α-helical secondary structure when bound to all bilayers.^7,8,12,13^ These measurements demonstrate that decreased activity of the macrolittins in diC20:1PC and in POPC+30% cholesterol is due to changes on their inherent nanopore-forming propensity and is not due to generic decreases in membrane binding or helicity. Thus, the results of our MD simulations are relevant to the interpretation of the membrane selectivity of the macrolittins.

Another important property of the nanopore-forming macrolittins is that they release small molecules and macromolecules from POPC with the same high potency.^10^ This means they *only form macromolecule-sized pores*. Alternately, MelP5, like most membrane permeabilizing peptides, forms small pores and releases small molecules at lower concentration and forms larger pores, releasing larger molecules, only at higher peptide concentration. The unexpected activity profile for M159 is shown by the peptide-induced release of the small molecules ANTS/DPX from bilayers made from POPC (Figure 5E). ANTS/DPX release by M159 occurs at roughly the same P:L as release of the macromolecule, TBD (Figure 5A). MelP5 on the other hand releases small molecules very potently, at P:L < 0.001, whereas it releases macromolecules at P:L > 0.01 (Figure 5A). In the ANTS/DPX release experiment in Figure 5E, M159, MelP5, and the three variants have similar permeabilizing activity in POPC. This means that the parents and variant sequences do not differ in their inherent membrane-permeabilizing activity in POPC for small molecules, which is very high. Instead, they differ specifically in *nanoporation activity*.

In POPC bilayers with 30% cholesterol, ANTS/DPX leakage caused by MelP5 is high, while ANTS/DPX leakage caused by M159 is lower (Figure 5F). For small molecule leakage from cholesterol containing bilayers, as shown earlier for TBD release, the three variants have activities that are intermediate to the two parents, despite the swapped relative activities of the parent molecules MelP5 and M159, relative to POPC. Taken together, the leakage data in Figure 5 show that the single residue changes in M159, 4EA, and 8EV specifically reduce macrolittin-like nanoportation but do not change overall membrane permeabilizing activity. This observation validates our use of MD simulations of macrolittins in POPC to understand the interactions that stabilize membrane-spanning nanopores.

### Vesicle Fusion: A Signature of Nanopore Formation by Macrolittins

**Figure 6:**
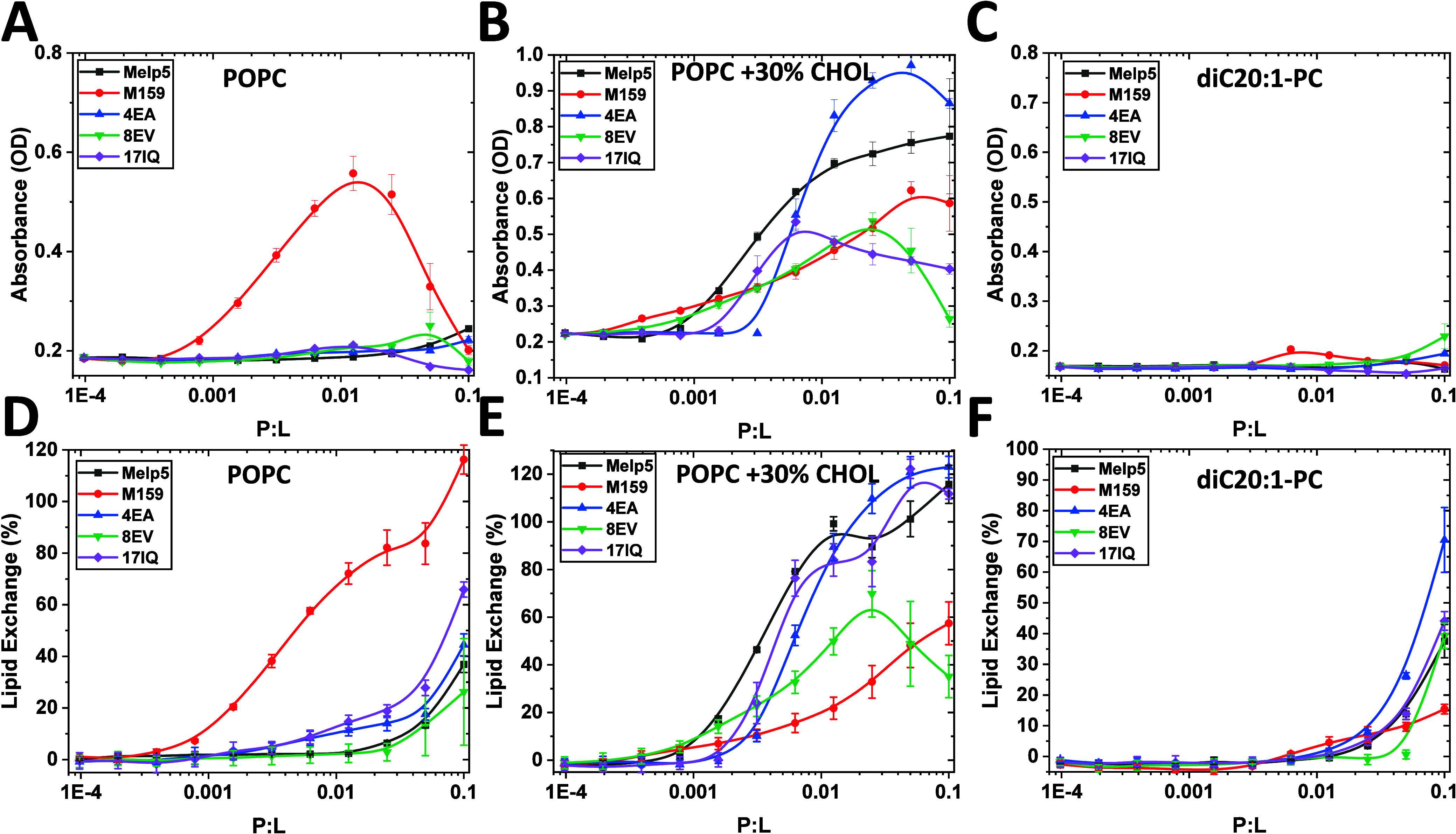
Peptide-induced aggregation and lipid exchange between vesicles. (A–C) Light scattering of vesicles consisting of 100% POPC (A); 70% POPC and 30% Cholesterol (B); 100% diC20:1-PC (C) incubated with peptides. 2 mM vesicles were incubated with peptides for 3 h at different peptide-to-lipid ratios. Optical absorbance was measured at 410 nm on a plate reader. (D–F) FRET between dual labeled vesicles and nonlabeled vesicles with the composition 100% POPC (D); 70% POPC and 30% cholesterol (E); 100% diC20:1-PC (F). 0.4 mM vesicles containing 0.5% NBD-PE and 0.5% rhodamine-PE dyes mixed with 2 mM vesicles were incubated with peptides for 3 h at different peptide-to-lipid ratios. NBD fluorescence was monitored on a plate reader, and lipid exchange percentage was calculated by the ratio of measured NBD fluorescence to NBD fluorescence from positive controls.

Previously, we showed that vesicle fusion at very low peptide concentration is a signature of nanopore formation by the macrolittins.^10^ We have speculated that the nanopore structure formed by the macrolittins exposes hydrophobic moieties that make the bilayers extremely prone to fusion. This idea is consistent with the multiple lipid molecules with highly distorted conformations observed in our simulations (Figures 2 and 3). Thus, to further explore membrane selectivity of the macrolittins and variants, we studied vesicle fusion by light scattering and by lipid exchange.^10^ In POPC bilayers (Figures 6A,D), M159 potently causes liposome fusion starting at concentrations as low as P:L = 0.001 while MelP5 causes little measurable fusion at concentrations up to P:L = 0.1. In this experiment, the three variants are indistinguishable from MelP5 in causing no measurable membrane fusion. These experiments show that the signature fusion activity of M159 in POPC is lost when even a single critical acidic residue is changed.

When the bilayers contain 30% cholesterol (Figure 6B,E), the fusion activity of M159 and MelP5 are reversed, with MelP5 having higher fusion activity and M159 much lower. Here again, the activities of the variants are intermediate to the activities of M159 and MelP5. In thicker C20:1PC bilayers where nanoporation does not occur, neither aggregation nor fusion are observed (Figure 6C,F), validating the correlation between nanopore formation and fusion activity.

### Cell Toxicity

**Figure 7:**
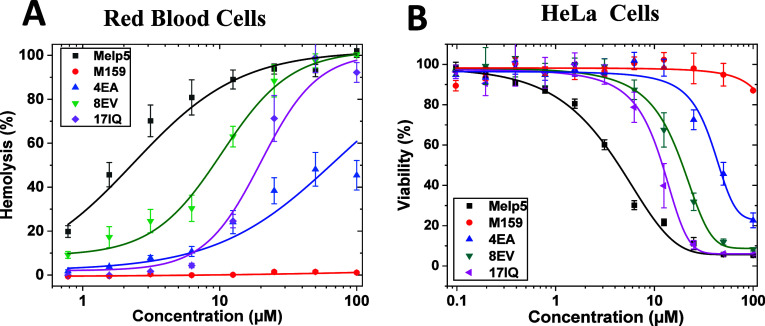
Toxicity of peptides on human cells and bacteria. (A) HeLa cells were incubated with different peptides shown at about 80% confluency for 3 h, then the cells were subject to cell toxicity assay. (B) Hemolysis of human red blood cells. Serially diluted peptides were incubated with human RBCs for 1 h. Release of hemoglobin was measured using optical absorbance of the cell supernatant at the heme absorbance wavelength of 410 nm, and % lysis against human erythrocytes is shown.

We also compared the cell toxicity of MelP5, M159, and the three variants using nucleated HeLa cells (Figure 7A) and human red blood cells (RBC, Figure 7B). This experiment tests the relation between the nanopore structure of the macrolittins, their high activity in POPC, and their low activity against cell membranes.^10^ HeLa cell cytotoxicity was measured by Alamar Blue, while RBC toxicity was measured by hemolysis. Melp5, like melittin, is highly toxic to HeLa cells at a low concentration of 3 μM. Despite its very high activity in POPC bilayers, M159 had almost no cellular toxicity even at a high concentration of 200 μM. The three variants showed intermediate toxicities, strongly supporting our hypothesis that these residues contribute importantly to membrane selectivity and thus to the lack of toxicity of the macrolittins. Accordingly, the two M159 variants E4A and E8V were significantly more toxic than M159 but less toxic than MelP5 (Figure 7A,B). MelP5 I17Q is less toxic than MelP5 but is more toxic than M159 or its variants. The 8EV variant, in particular, is at least 50-fold more toxic than M159. In agreement with these observations, RBC hemolysis measurements show that M159 E8V and E4V have increased hemolytic activity compared to M159, while I17Q has reduced hemolytic activity compared to MelP5 (Figure 7B). These results suggest that the membrane selectivity of the macrolittins is dependent on having the entire H-bond network intact.

### Structure of the Macrolittin Variants by MD Simulations

We next performed ∼500 ns MD simulations of the M159 variants E4A and E8V in POPC bilayers to compare with the M159 simulation described above. The starting configuration was the same as for the wild-type simulations shown in Figure 2. Time courses of direct and water-bridged H-bonds (Figure S10) show that the H-bond networks in the variants, like the parent peptide, are established quickly in the simulation and are stable. Due to the missing polar residues, the variants sample overall fewer total direct and water-mediated H-bonds during the simulations.

**Figure 8:**
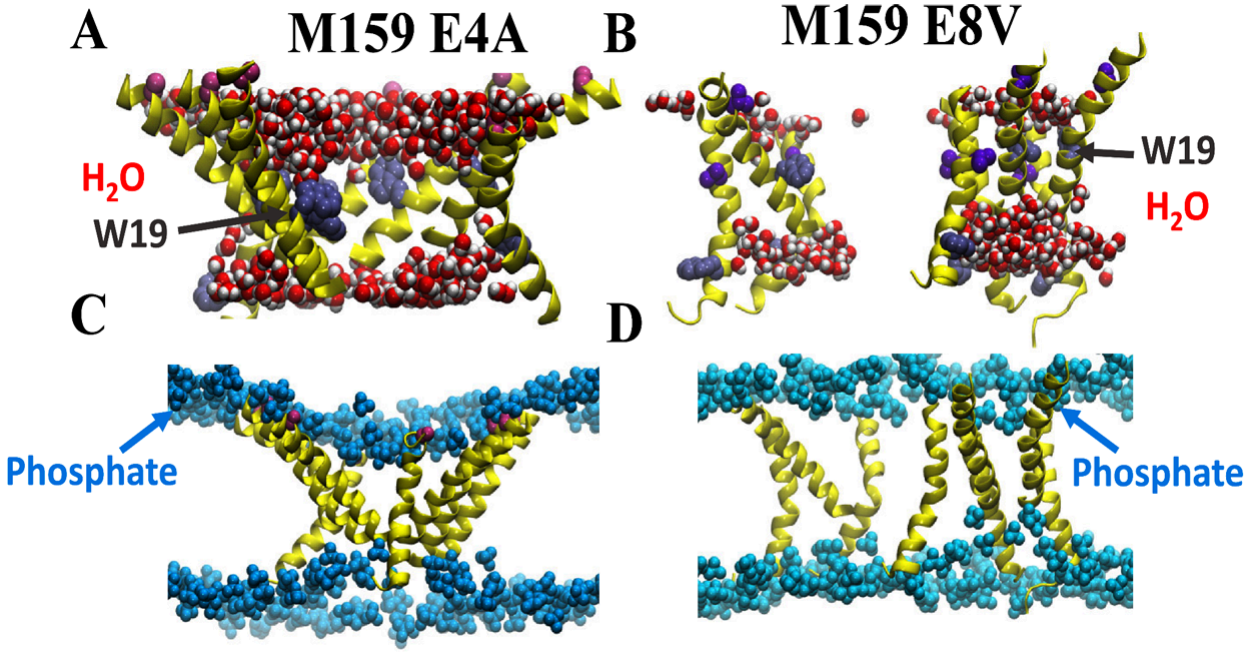
Lipid and water interactions inside macrolittin variant structures. The structure snapshots are from a 511 ns simulation of M159 E4A (A and C) and a 480 ns simulation M159 E8V (B and D). Peptides are shown in yellow. (A and B) Water molecules near the pore are shown in red and white spacefill. The residue W19 is shown in gray to indicate the depth of peptide in the bilayer. The varied residues E4A and E8V are shown in purple and violet, respectively. (C and D) Lipid phosphate groups are shown in blue. The left images are for macrolittin M159 E4A, while the right images are for M159 E8V.

Coordinate snapshots from the simulations of the variants are shown in Figure 8. There are informative differences between the variant structures and the wild-type macrolittin nanopore structures. First, the variant structures lack massive water penetration into the pore (Figure 8A,B), consistent with a role of carboxylic group hydration in pore forming. Second, the variant pore structures lack lipids with headgroups deep in the bilayer participating in the pore structure (Figure 8C,D) compared to Figures 2C,D. Third, on the time scale of our simulations the variant peptides can sample distinct membrane orientations compared to the parent sequence. For example, in Figure 8A,B, both the E4A and E8V variants have some of their Trp19 residues in the bilayer interface, some on the N-terminal side and some on the C-terminal side. We speculate that this movement of the variant peptides from their initially inserted location, with W19 deep in the bilayer, is an indication that the variant sequences have a propensity to deinsert from the transmembrane state into an interfacially bound state. This was not observed in simulations of M159 or M70.

**Figure 9:**
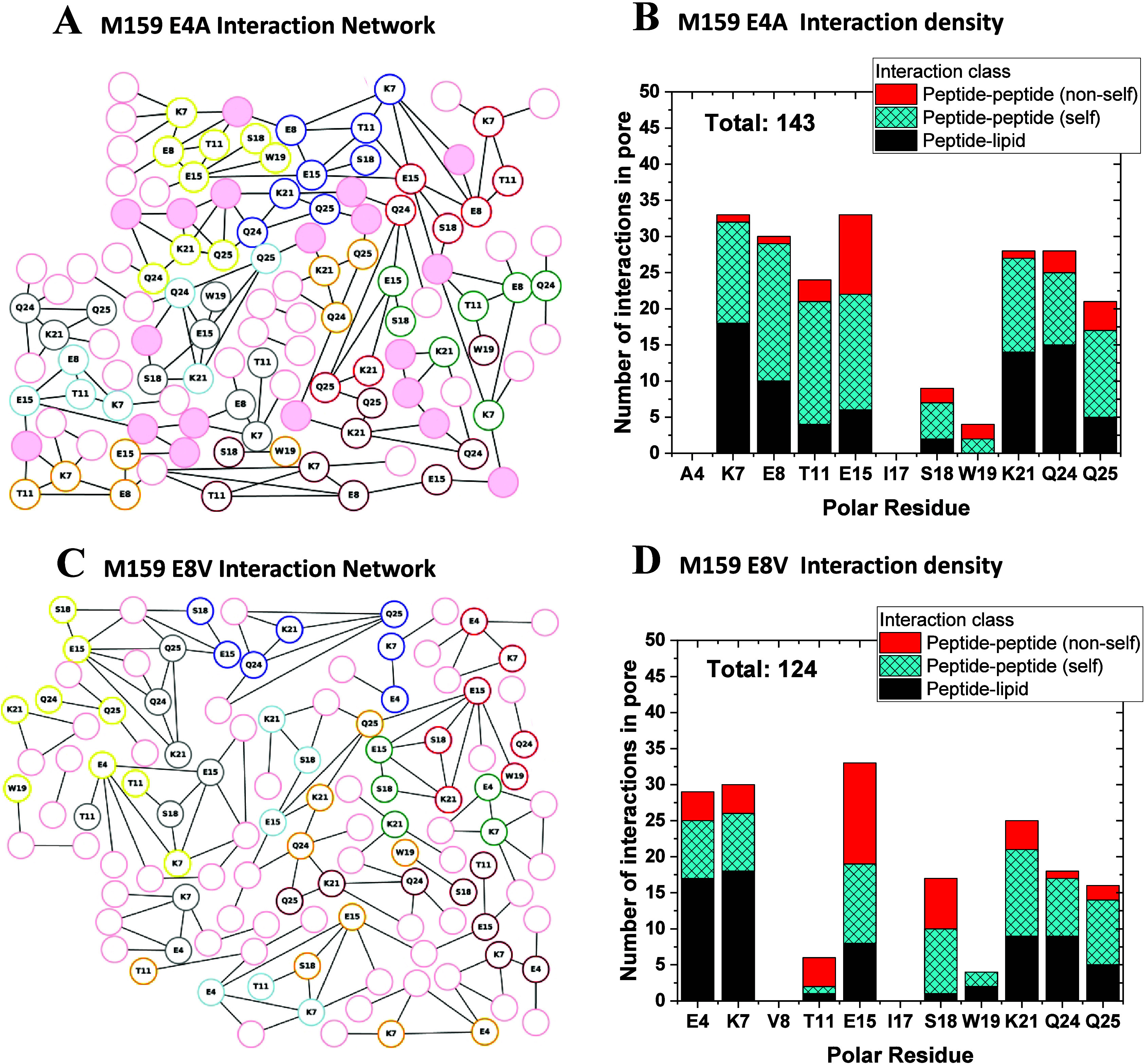
H-bond networks of the M159 E4A and M159 E8V macrolittins. H-bonds with up to three water bridges, and with at least 30% occupancy, were counted over the last 200 ns of each simulation. (A) H-bond graph of macrolittin M159 E4E after 480 ns simulation. Nodes representing lipid phosphate groups are shown as pink circles; full pink circles represent lipid phosphate groups that connect to two distinct peptides. Graph nodes colored other than pink represent peptide side chains. Lines (edges) between two graph nodes represent H-bonds, which can be direct or mediated by bridges of up to three H-bonding water molecules. Lipid nodes and edges that lack connections to peptide nodes, and only indicate lipid–lipid interactions, are excluded from the graph. (B) Number of interactions in the M159 E4A pore, presented as the sum of interactions per residue, for all eight peptides of the pore. (C) H-bond graph for the M159 E4A structure. (D) H-bond density for the M159 E8V structure.

In Figure 9 we show the H-bond interaction networks of the variants. In wild-type M159, both E4 and E8 of each peptide are critical contributors to the water-mediated H-bond network (Figure 4C,D). E4 and E8 have as many interactions as any other residue. E8 especially has intrahelical interactions with residues to which it has helical spacings, including E4 and T11, which are plus and minus one turn away, respectively, and E15, which is two turns away. E4 and E8 also interact with multiple lipid molecules each (Figure 9). When either E4 or E8 are replaced with a nonpolar residue, the H-bond network is significantly altered. It contains fewer total interactions, and overall, interactions with lipid phosphate groups are especially reduced. The parent M159 has 139 lipid interactions and M70 has 157, whereas M159 E4A has 108 and M159 E8V has 68 peptide–lipid direct and water bridged H-bonds. Whereas in the parent M159 peptide E4, E8, and K7 tend to sample intrapeptide H-bond bridges, with K7 additionally bridging (typically) to one or two lipid phosphate groups (Figure 4C), absence of the Glu side chain in E4A allows K7 to participate in more extensive local H-bond clusters with lipid phosphate groups (Figure 9A). Yet other arrangements of the local H-bond clusters that include K7 are found in the H-bond network of E8V (Figure 9C); here, E4 and K7 of one or two peptides can be part of small local H-bond clusters with nearby lipids (see red, blue, orange, brown, and gray nodes in Figure 9C) or participate in somewhat more extended interpeptide H-bond networks with E15 (see yellow, green, and cyan nodes in Figure 9C). That is, the local intrapeptide interactions within the two helical turns that host E4 and E8 shape the H-bond network interactions within the pore. As noted above for the parent M159, in both the E4A and E8V variants the K21, Q24, and Q25 triads tend to participate in intra- and interpeptide H-bond clusters with water and lipid molecules (Figure 9A,C).

To further test the robustness of these results, we performed repeat simulations of M159 E4A and M159 E8V in POPC bilayers lasting about 422 ns each. These repeat simulations were initiated from the same starting coordinates, but with different initial velocity assignment. Repeat simulations, described in detail Figures S10–S15, produce structures, H-bond networks, H-bond densities, and time courses that agree with the corresponding main simulations.

### Membrane Selectivity

The potency and membrane selectivity of macrolittin nanopore formation is very high. Of the thousands of known membrane permeabilizing peptides, few release small molecules at P:L < 1:1000,^8,10,19^ and only the closely related pHD peptides, at pH < 6, release *macromolecules* at the same low P:L as the macrolittins. Yet, the same peptides are inactive against mammalian and bacterial cell membranes^10^ and have poor activity against bilayers that are slightly thicker than POPC or bilayers containing cholesterol.^10^ The selectivity of the macrolittins for POPC is especially surprising given that POPC is a widely used physical chemical mimic of fluid phase mammalian cell membrane. For example, POPC bilayers have been used to mimic mammalian cell membranes in the study of antibacterial peptide selectivity for bacterial membranes.^20−22^ Integral membrane proteins have been shown to retain both structure and function when reconstituted into POPC bilayers.^23,24^ Most peptides that permeabilize POPC liposomes, including melittin and MelP5, readily permeabilize cell membranes as well.^19^

We have shown experimentally that macrolittins self-assemble into *membrane-spanning* α-helical nanopores even at very low peptide-to-lipid ratios.^9,12,13^ For membrane-spanning helical peptides, the macrolittins are unusually polar. The fact that their activity decreases, and only slightly, at pH 5 compared to pH 7 shows that the three acidic side chains are deprotonated and charged in the nanopore. Sliding window hydropathy scales^25^ predict that the macrolittins are far too polar, even if we assume the glutamates are protonated, to form membrane-spanning structures (Figure S16). Some transporter or ion channel membrane proteins also have a small number of such polar helices that span membranes,^26^ but these polar helices are stabilized significantly in the membrane by being topologically connected to multiple other very hydrophobic helices in the parent protein. Instead, the macrolittins in nanopores readily self-assemble into membrane-spanning nanopores from unconnected monomeric peptides without the stabilizing, entropic benefit of being connected to other nonpolar helices. *The macrolittin nanopore transmembrane structure is thus stabilized in an unusual, nonclassical manner.* In this work, we used MD simulations and experiments in synthetic and natural membranes to explore the structural basis for the unexpected stability and membrane selectivity of the macrolittins.

The MD simulations show that the macrolittin nanopore structure is dominated by a dense network of H-bonds that includes the array of polar residues on the polar surface of the helices. We note that this arrangement of polar and charged groups was specifically selected in the high-throughput screen for nanoporation. This network of H-bonds, which stabilizes the periphery of the large water-filled pores and spans the entire thickness of the nanopore across the membrane, includes many water and lipid molecules that bridge indirect peptide–peptide H-bonds. Over time, the simulations show that a single octameric pore will sample several hundred stabilizing polar interactions across the bilayer.

The macrolittin nanopore H-bond network also includes many lipid head groups, such that the network contains more lipid molecules than peptides, and many more peptide–lipid–water interactions than intermolecular peptide–peptide interactions (Figures 4 and 9). Highly distorted lipids, some oriented nearly parallel to the membrane surface, participate in the nanopore structure. Lipids are likely a critical contributing factor in nanopore stability. Such lipid-rich pore structures are examples of what have previously been called toroidal pores.^27,28^ The presence of such distorted lipids may explain why the nanopores form at such low concentration. The horizontally oriented acyl chains of these distorted lipids extend into the bilayer and help stabilize the peptide–membrane–water periphery. This would explain the sensitivity of nanopore formation to the addition of cholesterol which will dramatically alter the properties of the bilayer hydrocarbon phase and may make the horizontal acyl chains less likely to interact favorably with the membrane.

The three amino acid residues that we varied in this work, E4, E8 and Q17, were the most conserved of the polar and charged residues selected in the screens for the macrolittin and pHD peptides from a MelP5-based library.^7,8^ E4 and E8 especially contribute 30–40 stabilizing interactions each to the H-bond network, including intrapeptide, interpeptide, and peptide–lipid interactions.

We show here that the mutation of even a single one of these three polar residues has dramatic effects on the nanoporation activity of the macrolittins, including significantly decreased nanoporation, decreased vesicle fusion, decreased membrane selectivity, and increased cytotoxicity. Further, the simulations showed that the loss of E4 or E8 from the H-bond network reduced the total number of H-bonds, reduced the number of peptide–lipid interactions, reduced the number of lipids with highly distorted structures, and caused peptides to move from inserted toward interfacial locations in the membrane, consistent with their dramatic effects on observed nanoporation activity.

## Conclusions

Taken together, the atomistic simulations and experimental data show that the basis for the nanopore stability and membrane selectivity of the macrolittins is an extensive and highly cooperative network of water-bridged H-bonds between charged and polar side chains and lipid headgroups that spans the bilayer. Lipid molecules participate intimately in the pore structure and stabilization, possibly helping to explain the membrane selectivity of the macrolittins and their loss of cytotoxicity compared to the nonselective, cytotoxic parent, MelP5. This molecular understanding of nanopore stability can form the basis for many testable hypotheses and for additional generations of nanopore peptides that are optimized for specific membranes or specific applications.

## Methods

### MD Simulations and H-bond Graph Computations

Starting coordinates for the eight-macrolittin peptide systems were prepared using Phyre2,^30^ Chemistry at Harvard Molecular Mechanics (CHARMM),^31,32^ and Visual Molecular Dynamics (VMD).^33^ First, the coordinates for one macrolittin peptide were generated by using as a template for the peptide backbone the pHD105 peptide equilibrated in POPC. Second, seven additional copies of the peptide were generated and positioned relative to each other equidistantly, such that the starting pore diameter was compatible with the experimentally measured values for the smallest pores. The C-terminus of each peptide was treated as a neutral *N*-methylamide C-terminus. Using CHARMM-GUI,^34,35^ the peptides were inserted into hydrated POPC lipid membranes with 0.1 mM neutralizing NaCl salt concentration. Each simulation system contained ∼530 lipids and ∼26.740 water molecules. We used the TIP3P water model^36^ and the CHARMM36m parameters for protein, lipids, and ions.^37−40^ All simulations were performed using NAMD.^41−43^ Heating and early stages of equilibration with velocity rescaling were performed according to the standard CHARMM-GUI protocol^35^ and with an integration step of 1 fs and covalent bonds to H atoms kept fixed.^44^ Production runs were performed in the *NPT* ensemble (constant number of particles *N*, constant pressure *P* = 1.01 bar, and constant temperature *T* = 303.15K) using a Langevin dynamics scheme and a Nosé–Hoover piston.^45,46^ Short-range real space interactions were treated with a switch function between 10 and 12 Å and Coulomb interactions, with smooth particle mesh Ewald summation.^47,48^ During the first 1 ns an integration step of 1 fs was used, followed by a multiple time integration scheme^49^ with 1 fs for the bonded forces, 2 fs for short-range nonbonded, and 4 fs for long-range Coulomb interactions. Coordinates were saved each 10 ps. Production runs were prolonged as follows: the M159 wild-type variant, 496.9 ns; M159 E4A, 511.0 ns; M159 E8V, 480.9 ns; and M70, 511.0 ns. The total sampling time of the four main simulations we report is ∼2 μs.

For each simulation above, we further performed one repeat simulation that was initiated from the same starting coordinates, but with different initial velocity assignment. These replica simulations were prolonged as follows: the M159 wild-type variant, 411 ns; M159 E4A, 423 ns; M159 E8V, 428 ns; and M70, 422 ns, for a total sampling time of 1.67 μs.

Graphs of H-bond networks were computed using Bridge/Bridge2.^14,15^ Briefly, an H-bond graph consists of nodes (here, the H-bonding peptide side chains and lipid phosphate groups) and edges (here, direct or water-mediated H-bonds and bridges between the nodes of the graph. H-bonds were computed per residue using standard geometric criteria of distance ≤3.5 Å between the donor and acceptor heteroatoms and H-bond angle ≤60°. We included in the graph computations all H-bonding peptide side chains, phosphate groups of lipids that are within 15 Å of the pore’s peptide at the end of the simulations, and water-mediated H-bond bridges with up to three water molecules in the bridge.

The occupancy of an H-bond is given by the percentage of coordinate sets, used for data analyses, in which the H-bond criteria are met. H-bond graphs computed for the protein–water H-bond network of an unrelated soluble protein, PsbO, indicated that an occupancy threshold of 30% is reasonable to analyze side chain–side chain H-bonds and that a lower threshold of 20% is useful to evaluate more dynamic water-mediated connections.^50^ As a compromise between the need to include in analyses dynamic water-mediated bridges and the need for clarity of our H-bond graphs, which have numerous peptide and lipid H-bonding groups (nodes), we display all H-bond graphs at a minimum occupancy of 30%. Each graph was computed based on ∼20 000 equally spaced coordinate sets taken from the last 200 ns of the corresponding simulation. Additionally, from the complete production runs (i.e., excluding the initial equilibration phase) we report, for each of the four simulations and their corresponding repeats, the time series of the total number of direct and water-mediated bridges between side chains sampled.

### Materials

Peptides of >90% purity were synthesized by BioSynthesis, Inc. 1-Palmitoyl-2-oleoyl-*sn*-glycero-3-phosphocholine (POPC), 1-palmitoyl-2-oleoyl-*sn*-glycero-3-phospho-(1′-rac-glycerol) (POPG), 1,2-dimyristoleoyl-*sn*-glycero-3-phosphocholine (diC14:1PC), 1,2-dieicosenoyl-*sn*-glycero-3-phosphocholine (C20:1PC), phosphoethanolamine-N-(7-nitro-2-1,3-benzoxadiazol-4-yl) (NBD-PE), 1,2-dioleoyl-*sn*-glycero-3-phosphoethanolamine-N-(lissamine rhodamine B sulfonyl) (Rhodamine-PE), and cholesterol were purchased from Avanti Polar Lipids. 8-Aminonaphthalene-1,2,3-trisulfonic acid (ANTS) and *p*-xylylenebis (pyridinium bromide) (DPX) were purchased from Thermo Fisher Scientific. Chloroform, ammonium thiocyanate, and other salts and buffer materials were purchased from Fisher Scientific or Sigma-Aldrich. TAMRA-biotin-dextran (TBD) was synthesized as described elsewhere.

### Peptides

All peptides were synthesized by Biosynthesis Inc. and were validated by mass spectrometry and HPLC. Stock solutions of 1.2 mM peptides were prepared with 0.025% acetic acid. Concentrations were determined by measuring the absorbance of the single tryptophan on each peptide. The average of three absorbance measurements at 280 nm on a NanoDrop 2000c (Thermo Fisher Scientific) was used to calculate the concentration. Peptide powders were stored at −20 degrees until use, and peptide solutions were stored at 4 degrees.

### Liposome Preparation

Large unilamellar vesicles (LUV) of 100 nm diameter were prepared with different compositions of synthetic lipids. For vesicles without entrapped contents (empty vesicles) lipids in chloroform were dried under vacuum overnight, resuspended in pH 7 buffer (10 mM sodium phosphate, 100 mM KCl, pH 7), frozen and thawed ten times, and extruded over at least 10 times through 100 nm polycarbonate membranes. Empty vesicles were used for light scattering, lipid exchange, and vesicle fusion. For TBD-entrapping vesicles, dry lipid films were resuspended in buffer containing 1 mg of TBD per 50 μmol of lipid, and the solutions were subject to 10 freeze–thaw cycles using liquid nitrogen. After extrusion, vesicles were incubated on high-capacity streptavidin agarose to remove unencapsulated TBD. For ANTS/DPX vesicles, dried lipid films were resuspended in 12.5 mM ANTS and 45 mM DPX. Upon extrusion, unencapsulated ANTS and DPX were separated from the vesicles by size exclusion chromatography with Sephadex G-100 resin.

### Light Scattering Assays

2 mM liposomes with different lipid compositions were incubated with peptides for 3 h at peptide-to-lipid ratio (P:L) ranging from 1:10 to 1:10000 in 96-well plates, and as a negative control, no peptide was added to liposomes. Absorbance of liposome light scattering was measured at 410 nm by a Biotek Synergy plate reader (BioTek, USA). The measurements were repeated three times.

### Fluorescence Resonance Energy Transfer (FRET) Assays

0.4 mM Liposomes containing 0.5% NBD-PE and 0.5% rhodamine-PE dyes mixed with 1.6 mM pure POPC liposomes were incubated with Melp5 or M159 for 3 h at P:L ranging from 1:10 to 1:10000 in 96-well plates. NBD fluorescence was monitored on a plate reader (ex/em = 480/520 nm), and lipid exchange percentage was calculated by the ratio of measured NBD fluorescence to NBD fluorescence from positive controls (2 mM POPC liposomes containing 0.08% NBD-PE and 0.08% rhodamine-PE), and as a negative control, no peptide was added to mixed liposomes. The measurements were repeated three times. Fractional lipid exchange was calculated as
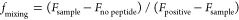
1

### Dextran Leakage Assays

Leakage of 40 kDa TBD was measured using Forster resonance energy transfer (FRET) as described. Dextran vesicles with entrapped TBD were diluted to 1 mM, and streptavidin-AF488 (the donor fluorophore) was added to a final concentration of 20 nM. In a 96-well plate, peptide and vesicles were mixed with P:L ranging from 1:10 to 1:10000 and then incubated while shaking at room temperature for 1 h before measuring FRET by donor fluorescence quenching with ex/em = 495/519 nm. As a positive control for 100% leakage, 4 μL of 10% Triton X100 was added to three wells, and as a negative control, no peptide was added to three wells. The measurements were repeated three times. Fractional leakage was calculated as
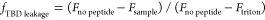
2

### ANTS/DPX Leakage Assays

Small-molecule leakage was measured by quenching ANTS with DPX. ANTS/DPX leakage vesicles were diluted to 1 mM. On a 96-well plate, peptide and vesicles were mixed at P:L ranging from 1:10 to 1:10000 and then incubated with shaking at room temperature for 1 h before measuring ANTS fluorescence using a microplate reader with ex/em = 360/519 nm. As a positive control for 100% leakage, 4 μL of 10% Triton X100 was added to three wells, and as a negative control, no peptide was added to three wells. The measurements were repeated three times. Fractional leakage was calculated as
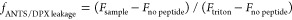
3

### Tryptophan Binding

100 μL of 10 μM Melp5 or M159 was prepared in HBS solutions in cuvettes. Liposomes with different lipid compositions were added with P:L ranging from 1:10 to 1:170. After 10 min of incubation at room temperature, tryptophan fluorescence spectra were measured on a spectrophotometer (HORIBA, Canada) and the peak fluorescence intensity was recorded at 333 nm (ex = 280 nm). To correct for lipid scattering,^51^ we measured fluorescence of free tryptophan at P:L ranging from 1:10 to 1:170. The fitting curve and mole-fraction partition coefficient, *K*_P_, was obtained by fitting using the equation
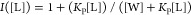
4where *K*_P_ is a mole-fraction partition coefficient, *I* is the fluorescence fold increase compared to no lipid binding, [L] is the lipid concentration, and [W] = 55.3 M is the molar concentration of water. The measurements were repeated three times.

### Cell Culture

HeLa cells were purchased from ATCC. Cells were cultured at 37 °C with 5% CO_2_ in Dulbecco’s Modified Eagle’s Medium (DMEM) (Gibco) supplemented with 10% fetal bovine serum (FBS) (Gibco), 1% antibiotic–antimycotic (Gibco), and 1% nonessential amino acids (Gibco). Cells were passaged 1:5 at 90% confluency.

### AlamarBlue Assays

Twenty-four hours after peptide or drug treatments, cells in 100 μL media were treated with 10 μL of 10× Alamar Blue reagent and incubated for 3 h at 37 °C. The fluorescence (ex570/em585) was measured and standardized to untreated wells.

### Human Serum and Erythrocytes

Fresh human serum (OTC) and human O+ erythrocytes were obtained from Interstate Blood Bank, Inc. RBCs were subjected to four cycles of centrifugation at 1000*g* with resuspension in fresh DPBS. Following the final wash step, the supernatant was clear and colorless. RBC concentration was determined using a hemocytometer.

### Hemolysis

Peptide was serially diluted in PBS starting at a concentration of 100 μM. An equal volume of RBCs in PBS at 2 × 10^8^ cells/mL was added. Controls were PBS only and 1% Triton. The mixtures were incubated at 37 °C for 1 h and centrifuged at 1000*g* for 5 min. The absorbance of released hemoglobin at 410 nm was recorded.

### Statistical Analyses

All data are presented as mean ± standard error (SE) of at least three independent biological experiments (*n* = 3). Graphs were drawn and statistical analyses were performed using GraphPad Prism or Origin.

## Supporting Information Available

Time course data for all molecular dynamics simulations. Molecular graphics images, H-bond networks and H-bond counts for all repeat simulations. Detailed plots of water bridge lengths and occupancy values for all simulations. Membrane binding measurements for all macrolittins. Circular dichroism spectra for all macrolittins. Hydropathy comparison of macrolittins and a known membrane spanning helix (PDF)

## References

[ref1] Guha S.; Ferrie R. P.; Ghimire J.; Ventura C. R.; Wu E.; Sun L.; Kim S. Y.; Wiedman G. R.; Hristova K.; Wimley W. C. Applications and evolution of melittin, the quintessential membrane active peptide. Biochem. Pharmacol. 2021, 193, 114769 10.1016/j.bcp.2021.114769. 34543656 PMC9235364

[ref2] Wimley W. C.; Hristova K. The Mechanism of Membrane Permeabilization by Peptides: Still an Enigma. Aust. J. Chem. 2020, 73 (3), 96–103. 10.1071/CH19449. PMC7185740 32341596

[ref3] Jallouk A. P.; Moley K. H.; Omurtag K.; Hu G.; Lanza G. M.; Wickline S. A.; Hood J. L. Nanoparticle incorporation of melittin reduces sperm and vaginal epithelium cytotoxicity. PLoS One 2014, 9 (4), e95411 10.1371/journal.pone.0095411. 24748389 PMC3991669

[ref4] Soman N. R.; Baldwin S. L.; Hu G.; Marsh J. N.; Lanza G. M.; Heuser J. E.; Arbeit J. M.; Wickline S. A.; Schlesinger P. H. Molecularly targeted nanocarriers deliver the cytolytic peptide melittin specifically to tumor cells in mice, reducing tumor growth. J. Clin. Invest 2009, 119 (9), 2830–2842. 10.1172/JCI38842. 19726870 PMC2735896

[ref5] Krauson A. J.; He J.; Wimley W. C. Gain-of-Function Analogues of the Pore-Forming Peptide Melittin Selected by Orthogonal High-Throughput Screening. J. Am. Chem. Soc. 2012, 134 (30), 12732–12741. 10.1021/ja3042004. 22731650 PMC3443472

[ref6] Krauson A. J.; Hall O. M.; Fuselier T.; Starr C. G.; Kauffman W. B.; Wimley W. C. Conformational Fine-Tuning of Pore-Forming Peptide Potency and Selectivity. J. Am. Chem. Soc. 2015, 137 (51), 16144–16152. 10.1021/jacs.5b10595. 26632653 PMC4697923

[ref7] Wiedman G.; Kim S. Y.; Zapata-Mercado E.; Wimley W. C.; Hristova K. PH-Triggered, Macromolecule-Sized Poration of Lipid Bilayers by Synthetically Evolved Peptides. J. Am. Chem. Soc. 2017, 139, 937–945. 10.1021/jacs.6b11447. 28001058 PMC5521809

[ref8] Li S.; Kim S. Y.; Pittman A. E.; King G. M.; Wimley W. C.; Hristova K. Potent Macromolecule-Sized Poration of Lipid Bilayers by the Macrolittins, A Synthetically Evolved Family of Pore-Forming Peptides. J. Am. Chem. Soc. 2018, 140 (20), 6441–6447. 10.1021/jacs.8b03026. 29694775

[ref9] Wiedman G.; Fuselier T.; He J.; Searson P. C.; Hristova K.; Wimley W. C. Highly efficient macromolecule-sized poration of lipid bilayers by a synthetically evolved peptide. J. Am. Chem. Soc. 2014, 136 (12), 4724–4731. 10.1021/ja500462s. 24588399 PMC3985440

[ref10] Sun L.; Hristova K.; Wimley W. C. Membrane-selective nanoscale pores in liposomes by a synthetically evolved peptide: implications for triggered release. Nanoscale 2021, 13 (28), 12185–12197. 10.1039/D1NR03084A. 34190297 PMC9265991

[ref11] He J.; Melnik L. I.; Komin A.; Wiedman G.; Fuselier T.; Morris C. F.; Starr C. G.; Searson P. C.; Gallaher W. R.; Hristova K. Ebola Virus Delta Peptide is a Viroporin. J. Virol 2017, e00438-17 10.1128/JVI.00438-17. 28539454 PMC5533898

[ref12] Kim S. Y.; Bondar A. N.; Wimley W. C.; Hristova K. pH-triggered pore-forming peptides with strong composition-dependent membrane selectivity. Biophys. J. 2021, 120 (4), 618–630. 10.1016/j.bpj.2021.01.010. 33460594 PMC7896028

[ref13] Kim S. Y.; Pittman A. E.; Zapata-Mercado E.; King G. M.; Wimley W. C.; Hristova K. Mechanism of Action of Peptides That Cause the pH-Triggered Macromolecular Poration of Lipid Bilayers. J. Am. Chem. Soc. 2019, 141 (16), 6706–6718. 10.1021/jacs.9b01970. 30916949

[ref14] Siemers M.; Lazaratos M.; Karathanou K.; Guerra F.; Brown L. S.; Bondar A.-N. Bridge: A graph-based algorithm to analyze dynamic H-bond networks in membrane proteins. J. Chem. Theory Comput. 2019, 15, 6781–6798. 10.1021/acs.jctc.9b00697. 31652399

[ref15] Siemers M.; Bondar A.-N. Interactive interface for graph-based analyses of dynamic H-bond networks: application to spike protein S. J. Chem. Inf. Model. 2021, 61, 2998–3014. 10.1021/acs.jcim.1c00306. 34133162

[ref16] Pino-Angeles A.; Lazaridis T. Effects of Peptide Charge, Orientation, and Concentration on Melittin Transmembrane Pores. Biophys. J. 2018, 114 (12), 2865–2874. 10.1016/j.bpj.2018.05.006. 29925023 PMC6026367

[ref17] Terwilliger T. C.; Eisenberg D. The structure of melittin. II. Interpretation of the structure. J. Biol. Chem. 1982, 257, 6016–6022. 10.1016/S0021-9258(20)65098-0. 7076662

[ref18] Rex S. A Pro --> Ala substitution in melittin affects self-association, membrane binding and pore-formation kinetics due to changes in structural and electrostatic properties. Biophys. Chem. 2000, 85 (2–3), 209–228. 10.1016/S0301-4622(00)00121-6. 10961508

[ref19] Guha S.; Ghimire J.; Wu E.; Wimley W. C. Mechanistic Landscape of Membrane-Permeabilizing Peptides. Chem. Rev. 2019, 119, 6040–6085. 10.1021/acs.chemrev.8b00520. 30624911 PMC9235363

[ref20] Oren Z.; Lerman J. C.; Gudmundsson G. H.; Agerberth B.; Shai Y. Structure and organization of the human antimicrobial peptide LL-37 in phospholipid membranes: relevance to the molecular basis for its non-cell-selective activity. Biochem. J. 1999, 341, 501–513. 10.1042/bj3410501. 10417311 PMC1220385

[ref21] Park S.; Jackman J. A.; Cho N. J. Comparing the Membrane-Interaction Profiles of Two Antiviral Peptides: Insights into Structure-Function Relationship. Langmuir 2019, 35 (30), 9934–9943. 10.1021/acs.langmuir.9b01052. 31291111

[ref22] Chen C. H.; Starr C. G.; Troendle E.; Wiedman G.; Wimley W. C.; Ulmschneider J. P.; Ulmschneider M. B. Simulation-Guided Rational de Novo Design of a Small Pore-Forming Antimicrobial Peptide. J. Am. Chem. Soc. 2019, 141 (12), 4839–4848. 10.1021/jacs.8b11939. 30839209

[ref23] Liko I.; Degiacomi M. T.; Lee S.; Newport T. D.; Gault J.; Reading E.; Hopper J. T. S.; Housden N. G.; White P.; Colledge M.; et al. Lipid binding attenuates channel closure of the outer membrane protein OmpF. Proc. Natl. Acad. Sci. U. S. A. 2018, 115 (26), 6691–6696. 10.1073/pnas.1721152115. 29891712 PMC6042154

[ref24] Isele J.; Sakmar T. P.; Siebert F. Rhodopsin activation affects the environment of specific neighboring phospholipids: an FTIR spectroscopic study. Biophys. J. 2000, 79 (6), 3063–3071. 10.1016/S0006-3495(00)76541-6. 11106612 PMC1301183

[ref25] Snider C.; Jayasinghe S.; Hristova K.; White S. H. MPEx: a tool for exploring membrane proteins. Protein Sci. 2009, 18 (12), 2624–2628. 10.1002/pro.256. 19785006 PMC2821280

[ref26] Guan L.; Kaback H. R. Lessons from lactose permease. Annu. Rev. Biophys. Biomol. Struct. 2006, 35, 67–91. 10.1146/annurev.biophys.35.040405.102005. 16689628 PMC2802108

[ref27] Allende D.; Simon S. A.; McIntosh T. J. Melittin-induced bilayer leakage depends on lipid material properties: evidence for toroidal pores. Biophys. J. 2005, 88 (3), 1828–1837. 10.1529/biophysj.104.049817. 15596510 PMC1305237

[ref28] Pokorny A.; Almeida P. F. Kinetics of dye efflux and lipid flip-flop induced by delta-lysin in phosphatidylcholine vesicles and the mechanism of graded release by amphipathic, alpha-helical peptides. Biochemistry 2004, 43 (27), 8846–8857. 10.1021/bi0497087. 15236593

[ref29] Thornig P. JURECA: Data Centric and Booster Modules implementing the Modular Supercomputing Architecture at Jülich Supercomputing Centre. Journal of Large Scale Research Facilities 2021, 7, A182 10.17815/jlsrf-7-182.

[ref30] Kelley L. A.; Mezulis S.; Yates C. M.; Wass M. N.; Sternberg M. J. E. The Phyre2 web portal for protein modeling, prediction and analysis. Nat. Protoc. 2015, 10, 845–857. 10.1038/nprot.2015.053. 25950237 PMC5298202

[ref31] Brooks B. R.; Bruccoleri R. E.; Olafson B. D.; States D. J.; Swaminathan S.; Karplus M. CHARMM: a program for macromolecular energy, minimization, and dynamics calculations. J. Comput. Chem. 1983, 4, 187–217. 10.1002/jcc.540040211.

[ref32] Brooks B. R.; Brooks C. L. I.; MacKerell A. D. Jr.; Nilsson L.; Petrella R. J.; Roux B.; Won Y.; Archontis G.; Bartels C.; Boresch S.; et al. CHARMM: the biomolecular simulation program. J. Comput. Chem. 2009, 30, 1545–1614. 10.1002/jcc.21287. 19444816 PMC2810661

[ref33] Humphrey W.; Dalke W.; Schulten K. VMD: visual molecular dynamics. J. Mol. Graph. 1996, 14, 33–38. 10.1016/0263-7855(96)00018-5. 8744570

[ref34] Wu E. L.; Cheng X.; Jo S.; Rui H.; Song K. C.; Dávila-Contreras E. M.; Qi Y.; Lee J.; Monje-Galvan V.; Venable R. M.; et al. CHARMM-GUI Membrane Builder toward realistic biological membrane simulations. J. Comput. Chem. 2014, 35, 1997–2004. 10.1002/jcc.23702. 25130509 PMC4165794

[ref35] Lee J.; Cheng X.; Swails J. M.; Yeom M. S.; Eastman P. K.; Lemkul J. A.; Wei S.; Buckner J.; Jeong J. C.; Qi Y.; et al. CHARMM-GUI input generator for NAMD, GROMACS, AMBER, OpenMM, and CHARMM/OpenMM Simulations using the CHARMM36 additive force field. J. Chem. Theory Comput. 2016, 12, 405–413. 10.1021/acs.jctc.5b00935. 26631602 PMC4712441

[ref36] Jorgensen W. L.; Chandrasekhar J.; Madura J. D.; Impey R. W.; Klein M. L. Comparison of simple potential functions for simulating liquid water. J. Chem. Phys. 1983, 79, 926–935. 10.1063/1.445869.

[ref37] Klauda J. B.; Venable R. M.; Freites J. A.; O’Connor J. W.; Tobias D. J.; Mondragon-Ramirez C.; Votrobyov I.; MacKerell A. D. Jr.; Pastor R. W. Update of the CHARMM all-atom additive force field for lipids: validation on six lipid types. J. Phys. Chem. B 2010, 114, 7830–7843. 10.1021/jp101759q. 20496934 PMC2922408

[ref38] MacKerell A. D. Jr.; Feig M.; Brooks C. L. I. Extending the treatment of backbone energetics in protein force fields: limitations of gas-phase quantum mechanics in reproducing protein conformational distributions in molecular dynamics simulations. J. Comput. Chem. 2004, 25, 1400–1415. 10.1002/jcc.20065. 15185334

[ref39] Pastor R. W.; MacKerell A. D. Jr. Development of the CHARMM force field for lipids. J. Phys. Chem. Lett. 2011, 2, 1526–1532. 10.1021/jz200167q. 21760975 PMC3133452

[ref40] Venable R. M.; Luo Y.; Gawrisch K.; Roux B.; Pastor R. W. Simulations of anionic lipid membranes: development of interaction-specific ion parameters and validation using NMR data. J. Phys. Chem. B 2013, 117, 10183–10192. 10.1021/jp401512z. 23924441 PMC3813009

[ref41] Phillips J. C.; Braun B.; Wang W.; Gumbart J.; Takjkhorshid E.; Villa E.; Chipot C.; Skeel R. D.; Kale L.; Schulten K. Scalable molecular dynamics with NAMD. J. Comput. Chem. 2005, 26, 1781–1802. 10.1002/jcc.20289. 16222654 PMC2486339

[ref42] Phillips J. C.; Hardy D. J.; Maia J. D. C.; Stone J. E.; Ribeiro J. V.; Bernardi R. C.; Buch R.; Fiorin G.; Henin J.; Jiang W.; et al. Scalable molecular dynamics on CPU and GPU architectures with NAMD. J. Chem. Phys. 2020, 153, 044130 10.1063/5.0014475. 32752662 PMC7395834

[ref43] Kalé L.; Skeel R.; Bhandarkar M.; Brunner R.; Gursoy A.; Krawetz N.; Phillips J.; Shinozaki A.; Varadarajan K.; Schulten K. NAMD2: greater scalability for parallel molecular dynamics. J. Comput. Phys. 1999, 151, 283–312. 10.1006/jcph.1999.6201.

[ref44] Ryckaert J.-P.; Ciccotti G.; Berendsen H. J. C. Numerical integration of the Cartesian equations of motion of a system with constraints. Molecular dynamics of n-alkanes. J. Comput. Phys. 1977, 23, 327–341. 10.1016/0021-9991(77)90098-5.

[ref45] Martyna G. J.; Tobias D. J.; Klein M. L. Constant-pressure molecular-dynamics algorithms. J. Chem. Phys. 1994, 101, 4177–4189. 10.1063/1.467468.

[ref46] Hoover W. G. Canonical dynamics: equilibrium phase-space distribution. Phys. Rev. A 1985, 31, 1695–1697. 10.1103/PhysRevA.31.1695. 9895674

[ref47] Darden T.; York D.; Pedersen L. Particle mesh Ewald: an N x log(N) method for Ewald sums in large systems. J. Chem. Phys. 1993, 98, 10089–10092. 10.1063/1.464397.

[ref48] Essmann U.; Perera L.; Berkowitz M. L.; Darden T.; Lee H.; Pedersen L. G. A smooth particle mesh Ewald method. J. Chem. Phys. 1995, 103, 8577–8593. 10.1063/1.470117.

[ref49] Tuckerman M.; Berne B. J.; Martyna G. J. Reversible multiple time scale molecular dynamics. J. Chem. Phys. 1992, 97, 1990–2001. 10.1063/1.463137.

[ref50] Bondar A.-N. Interplay between local protein interactions and water bridging of a proton antenna carboxylate cluster. BBA - Biomembranes 2022, 1864, 184052 10.1016/j.bbamem.2022.184052. 36116514

[ref51] Ladokhin A. S.; Jayasinghe S.; White S. H. How to measure and analyze tryptophan fluorescence in membranes properly, and why bother?. Anal. Biochem. 2000, 285 (2), 235–245. 10.1006/abio.2000.4773. 11017708

