# Supplementary material for: Structural Determinants of Peptide Nanopore Formation
Source: ACS Nano. 2024 Jun 6;18(24):15831–44. doi: 10.1021/acsnano.4c02824 (PMC11191747; doi:10.1021/acsnano.4c02824)
Supplement: Supplementary file 1 — nn4c02824_si_001.pdf [file nn4c02824_si_001.pdf]

SUPPLEMENTAL Information

Structural Determinants of Peptide Nanopore Formation

Leisheng Sun[1,2], Kalina Hristova[3,4], Ana-Nicoleta Bondar,[5,6] William C. Wimley[1]

[1]Department of Biochemistry and Molecular Biology, Tulane University School of Medicine, New Orleans, LA 70112

[2]Current address: Key Laboratory of Study and Discovery of Small Targeted Molecules of Hunan Province, Department of Pharmacy, School of Medicine, Hunan Normal University, Changsha, 410013, China

[3]Department of Materials Science and Engineering, Whiting School of Engineering, Johns Hopkins University, Baltimore MD, 21218

[4]Institute for NanoBioTechnology, Johns Hopkins University, Baltimore, Maryland 21218

[5]University of Bucharest, Faculty of Physics, Atomiștilor 405, Măgurele 077125, Romania

[6]Forschungszentrum Jülich, Institute of Computational Biomedicine, IAS-5/INM-9, Wilhelm-Johnen Straße, 5428 Jülich, Germany

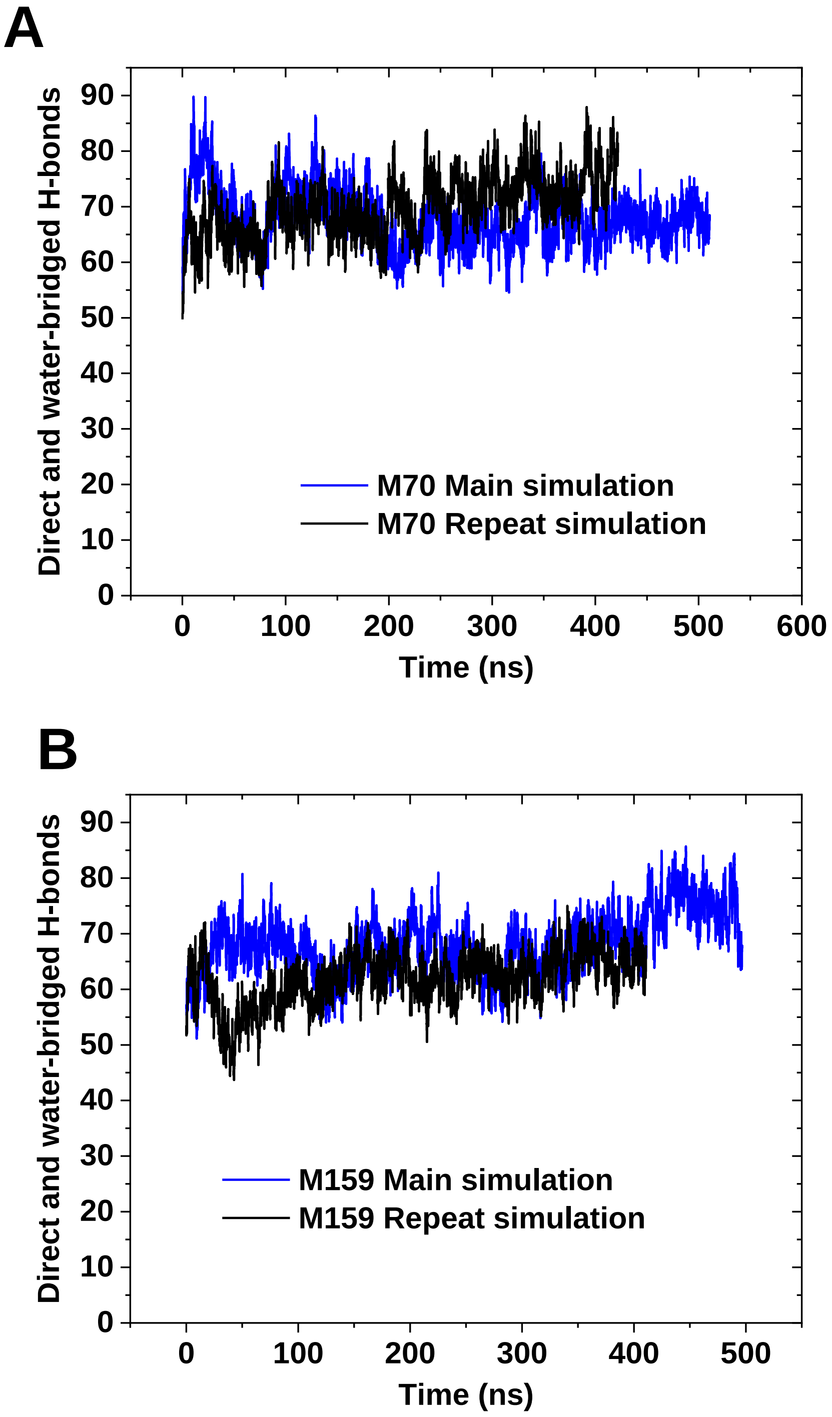

Figure S1. Time courses of the number of direct and water-mediated H-bonding of M70 and M159 during the production runs of the simulations. We present the total number of direct H-bonds between peptide sidechains (self and non-self), and water-mediated bridges between sidechains with up to three H-bonded water molecules per bridge. All H-bonds sampled at least once, regardless of the average H-bond occupancies, are included in these time series. The origin of time is the start of the production runs without any constraints. Black and blue profiles represent time courses computing from the main and repeat simulations, respectively. (A, B) Time series computed for macrolittin M70 (panel A) and M159 (panel B).

M70 Repeat Simulation

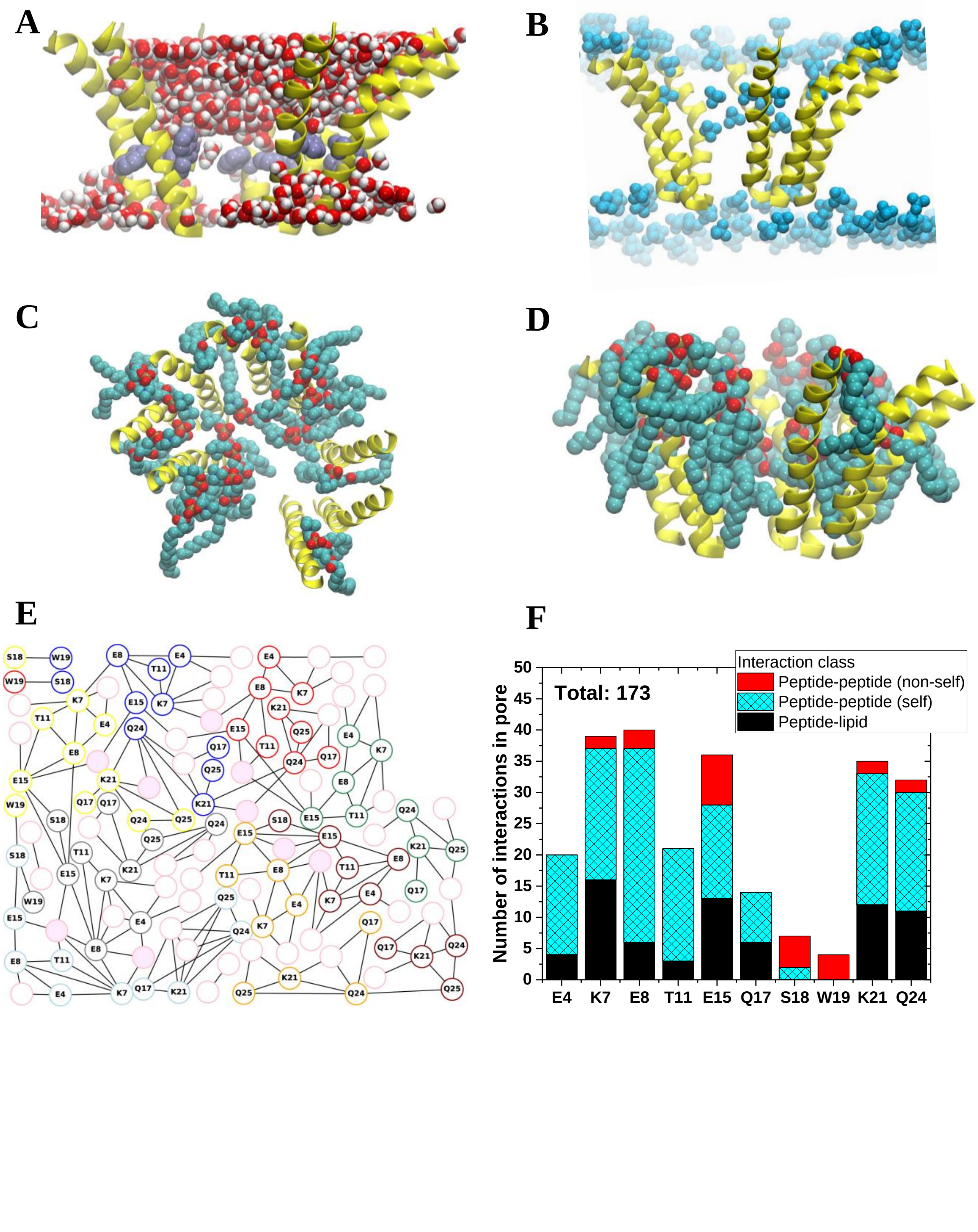

Figure S2. Results of repeat simulation of macrolittin M70. (A) Cut-away views showing water molecules interacting in the M70 pore. Peptides are shown as yellow ribbons, and water molecules are shown as van der Waals spheres with oxygen and H atoms colored red and white, respectively. The W19 sidechain of each peptide is shown as van der Waals spheres; for clarity, H atoms of the W19 sidechains are not shown. (B) Cut-away view illustrating lipid phosphate groups. (C, D) Illustration of lipid molecules closely associated with the M70 pore. In panel C, the pore is viewed from the membrane surface, while panel D shows a lateral view of the pore. Note that, for clarity, lipids of only one membrane leaflet are shown; see panel B for the phosphate groups of both membrane leaflets. (E, F) H-bond graph computed from the last ~200ns of the repeat simulation of macrolittin M70. For clarity, we include only H-bonds with a minimum occupancy of 30%. Detailed information on the average occupancy and average number of water molecules for each edge of the graph is presented in Figure S4. (F) Summary of the number of H-bonds counted based on the H-bond graph presented in panel E.

M159 Repeat Simulation

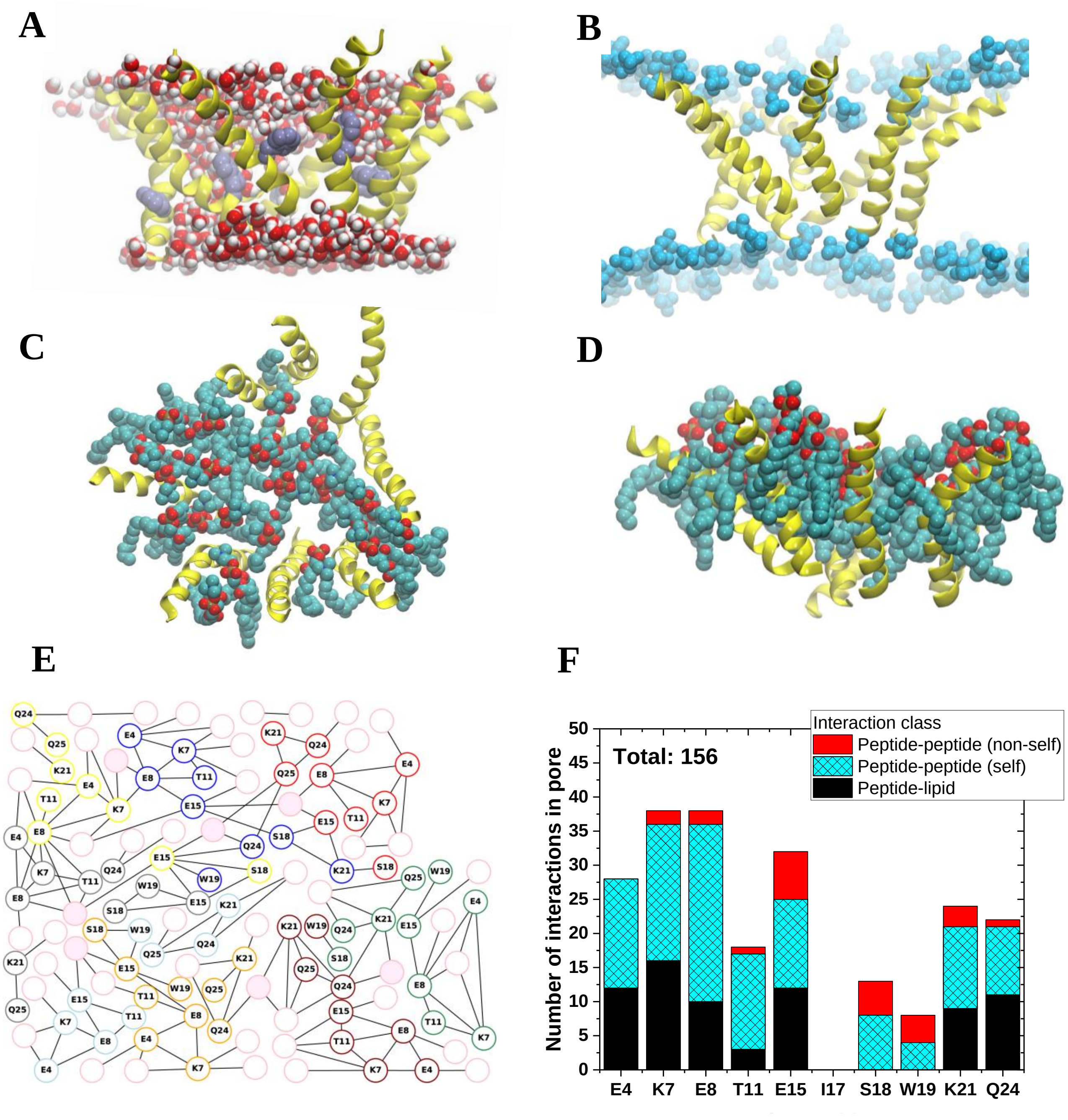

Figure S3. Results of repeat simulation of macrolittin M159. (A) Cut-away views showing water molecules interacting in the M159 pore. Peptides are shown as yellow ribbons, and water molecules are shown as van der Waals spheres with oxygen and H atoms colored red and white, respectively. The W19 sidechain of each peptide is shown as van der Waals spheres; for clarity, H atoms of the W19 sidechains are not shown. (B) Cut-away view illustrating lipid phosphate groups. (C, D) Illustration of lipid molecules closely associated with the M159 pore. In panel C, the pore is viewed from the membrane surface, while panel D shows a lateral view of the pore. Note that, for clarity, lipids of only one membrane leaflet are shown; see panel B for the phosphate groups of both membrane leaflets. (E, F) H-bond graph computed from the last ~200ns of the repeat simulation of macrolittin M159. For clarity, we include only H-bonds with a minimum occupancy of 30%. Detailed information on the average occupancy and average number of water molecules for each edge of the graph is presented in Figure S7. (F) Summary of the number of H-bonds counted based on the H-bond graph presented in panel E.

M70 Main Simulation

Average waters per bridge

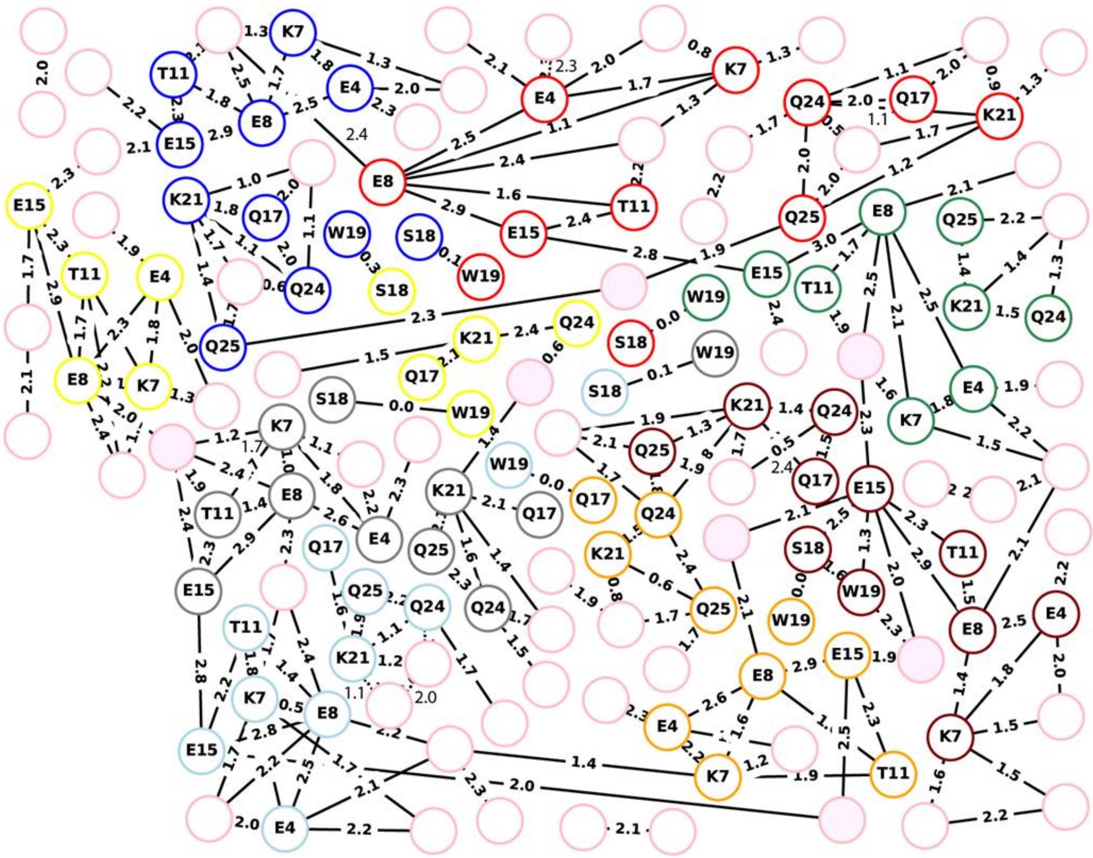

Average % occupancy

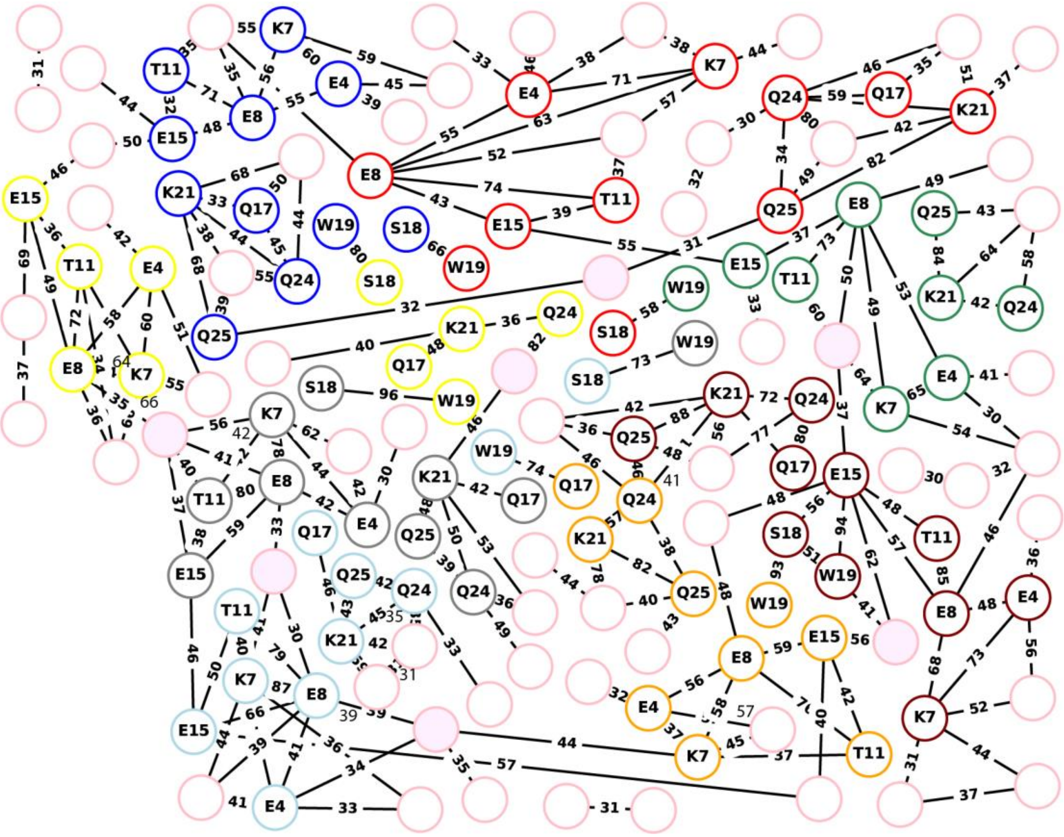

Figure S4. Results of the main simulation of macrolittin M70. These images display the average number of water molecules in the H-bonded bridges (top) and the average % occupancy of each direct or water bridged H-bond (bottom). Values were computed from the last ~200ns of the main simulation of macrolittin M70. For clarity, we include only H-bonds with a minimum occupancy of 30%.

M70 Repeat Simulation

Average waters per bridge

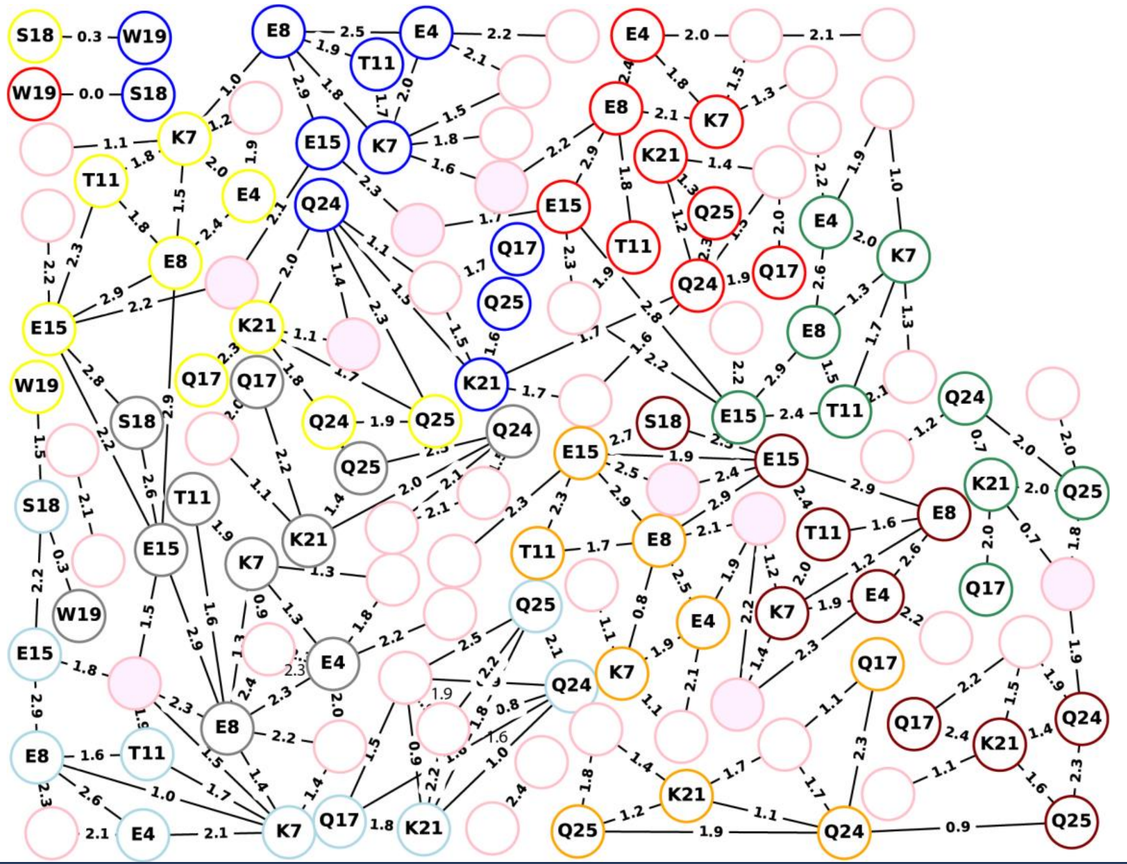

Average % occupancy

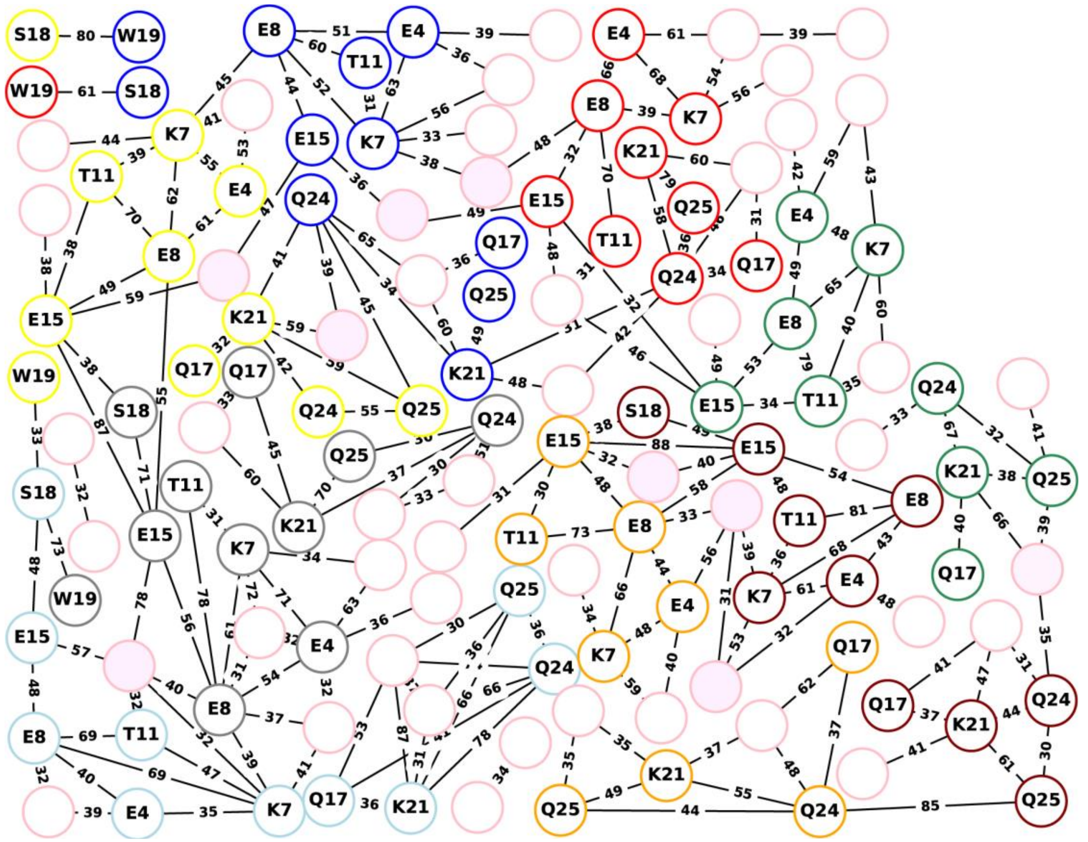

Figure S5. Results of the repeat simulation of macrolittin M70. These images display the average number of water molecules in the H-bonded bridges (top) and the average % occupancy of each direct or water bridged H-bond (bottom). Values were computed from the last ~200ns of the repeat simulation of macrolittin M70. For clarity, we include only H-bonds with a minimum occupancy of 30%.

M159 Main Simulation

Average waters per bridge

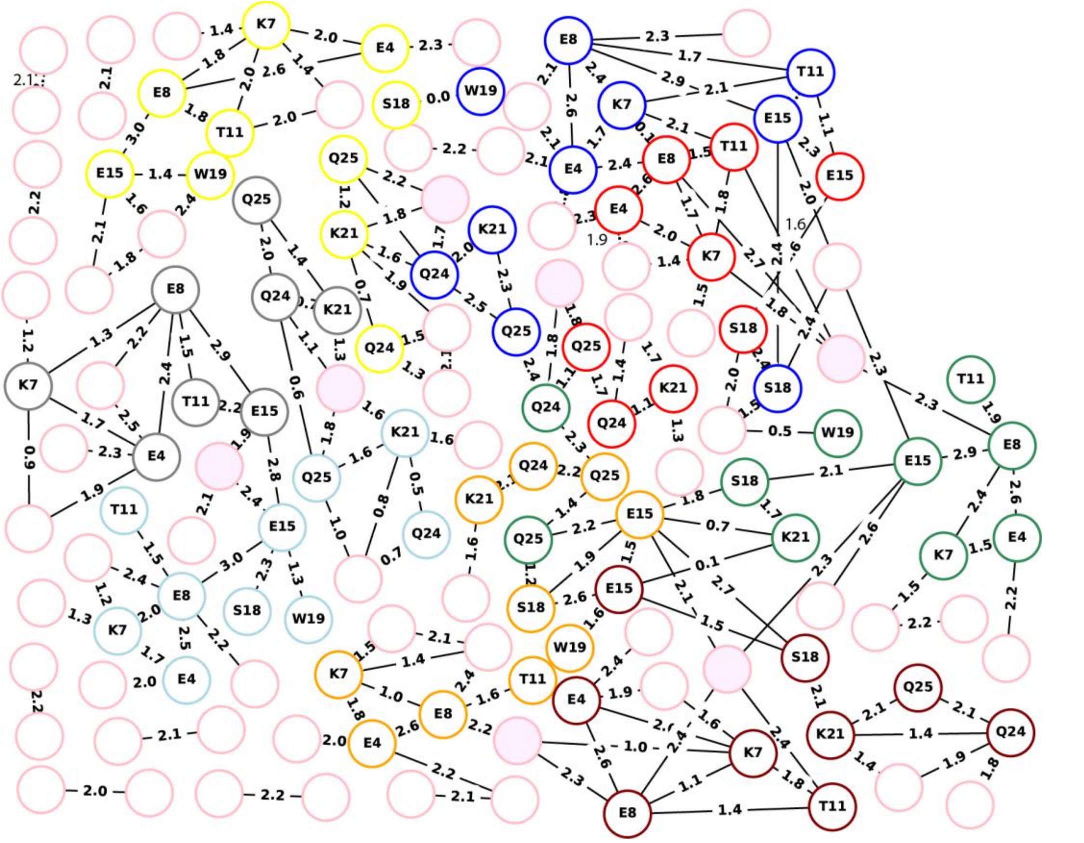

Average % occupancy

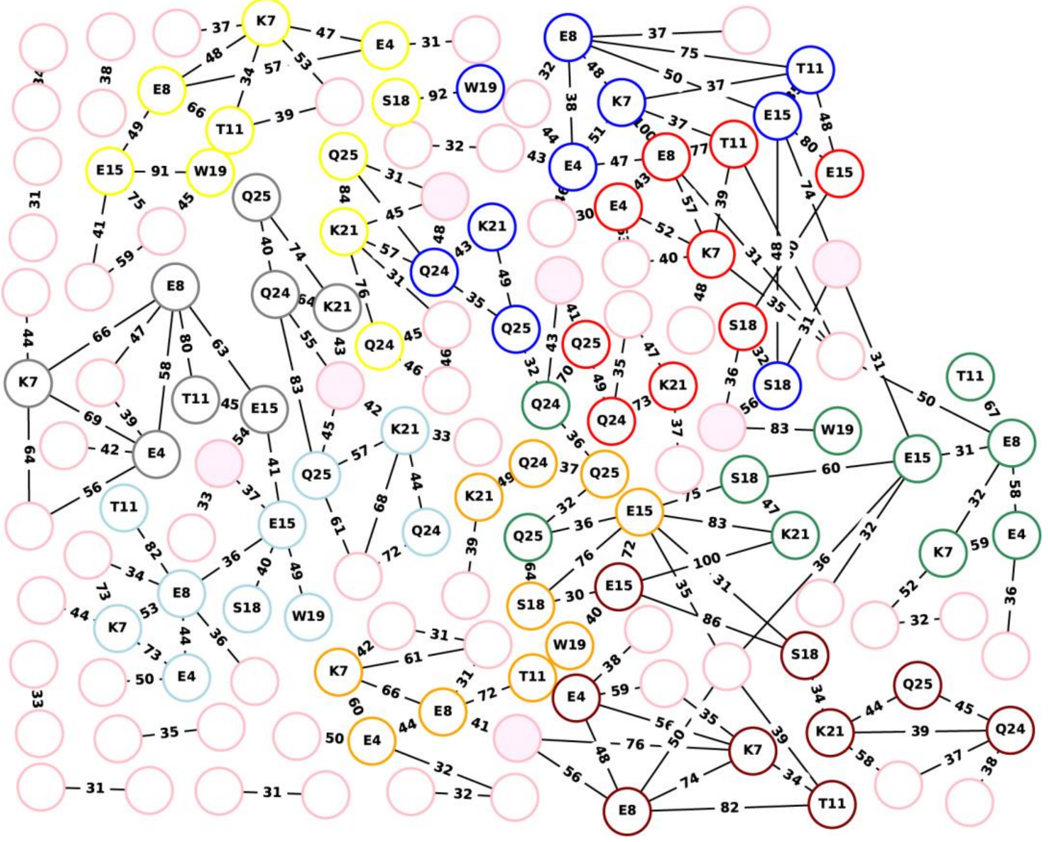

Figure S6. Results of the main simulation of macrolittin M159. These images display the average number of water molecules in the H-bonded bridges (top) and the average % occupancy of each direct or water bridged H-bond (bottom). Values were computed from the last ~200ns of the main simulation of macrolittin M159. For clarity, we include only H-bonds with a minimum occupancy of 30%.

M159 Repeat Simulation

Average waters per bridge

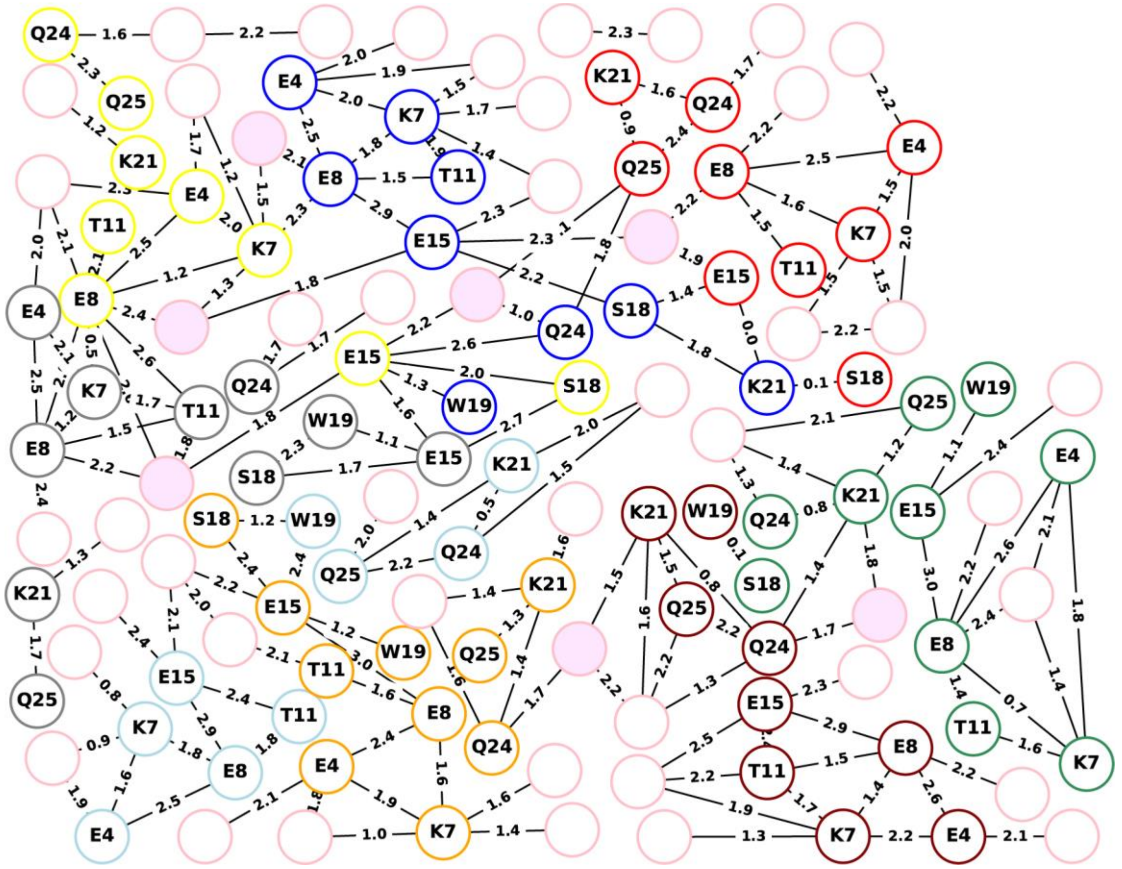

Average % occupancy

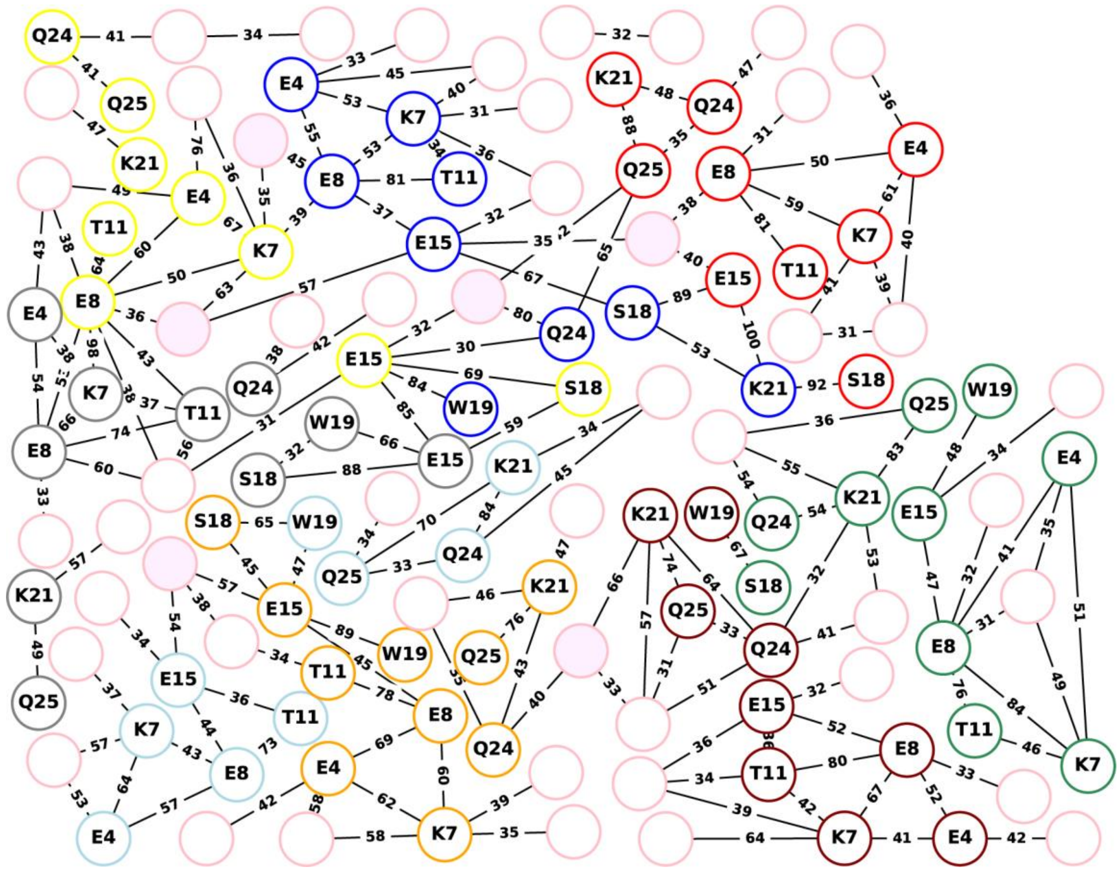

Figure S7. Results of the repeat simulation of macrolittin M159. These images display the average number of water molecules in the H-bonded bridges (top) and the average % occupancy of each direct or water bridged H-bond (bottom). Values were computed from the last ~200ns of the repeat simulation of macrolittin M159. For clarity, we include only H-bonds with a minimum occupancy of 30%.

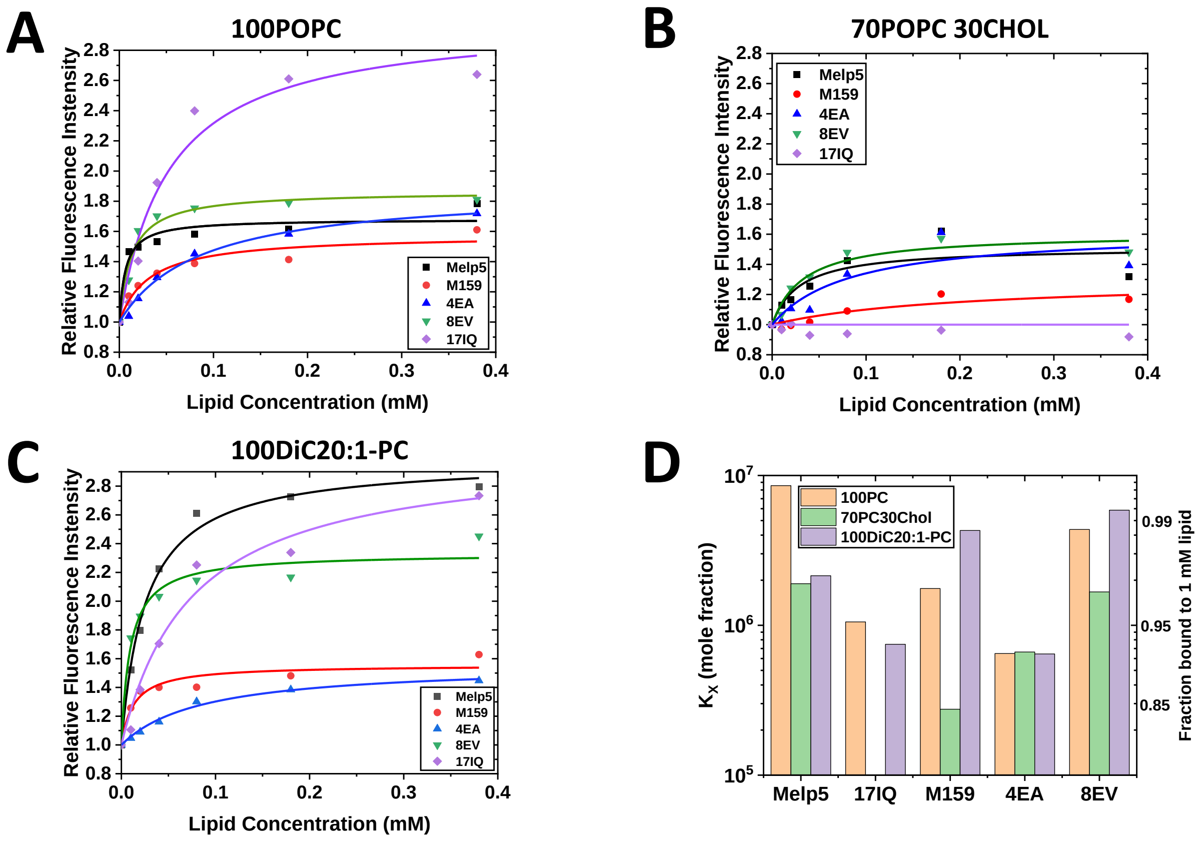

Figure S8. Membrane binding of macrolittins and variants. **A-C.** 10 µM peptides were titrated with lipid vesicles, and the tryptophan fluorescence spectra were measured. The fluorescence intensity at 333 nm was measured for each lipid concentration and was divided by the intensity measured in the absence of lipid. The intensity increase indicates membrane binding, and the more fraction partition coefficient Kx can be determined from these curves by Eq. 4 main text. Binding was measured to vesicles of 100% POPC (**A**); 70% POPC and 30% Cholesterol (**B**); 100% diC20:1-PC (**C**). **D.** The measured K_X values are shown in this panel on the left axis. On the right axis, we calculate the fraction of peptide bound to 1 mM lipid vesicles, as used in leakage, fusion and circular dichroism experiments. The macrolittins and their variants bind well to these lipid vesicles with 85-99% bound under typical conditions. The only exception is MelP5 I17Q, which does not bind measurably to POPC+30% cholesterol.

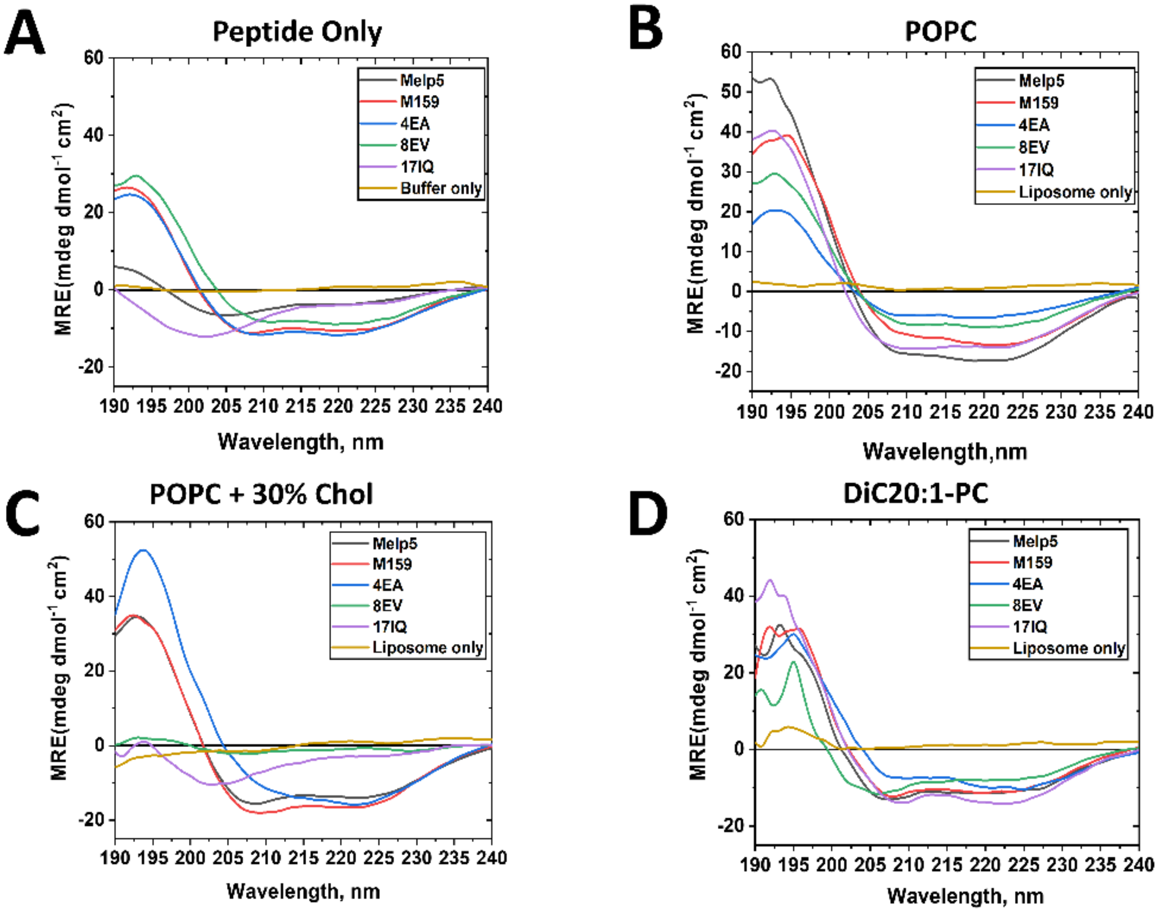

Figure S9. Circular dichroism spectra of macrolittins and variants. **A.** 25 µM of each peptide in buffer was characterized at room temperature using a JASCO 810 CD spectrometer. **C-D**. Circular dichroism spectra were collected in samples containing 25 µM peptide plus 1 mM of lipid vesicles made from POPC (**B**), POPC+30% cholesterol (**C**) and diC20:1PC (**D**). Ellipticity were converted to mean residues ellipticity. Maximum helicity for this family of peptides corresponds to about -20 mdeg $dmol^{-1}$ cm^{-1}. As shown in Fig, S4, the macrolittins and their variants bind well to these lipid vesicles with 85-99% bound under typical conditions. The only exception is MelP5 I17Q, which does not bind measurably to POPC+30% cholesterol. These data show that the macrolittins are their variants are α-helical when bound to these various bilayers. The only exception is MelP5 I17Q which is not helical in the presence of POPC+30% cholesterol. This agrees with the conclusion that MelP5 I17Q does not bind to cholesterol-containing bilayers. Most importantly, these data show that the M159 E4A and E8V variants bind strongly (**Fig. S4**) and have helical secondary structure in POPC bilayers. Their decrease in nanopore forming activity is due to a decrease in nanopore stabilizing interactions.

A

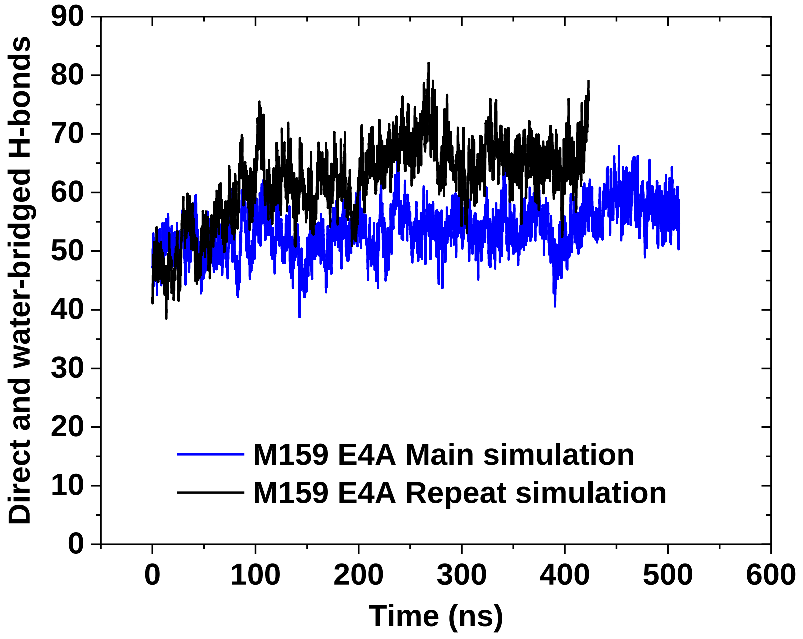

B

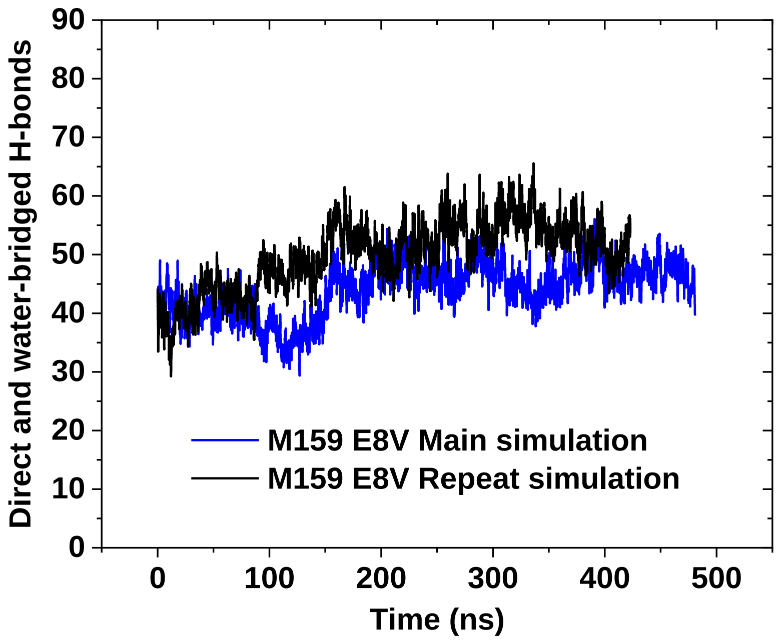

Figure S10. Time courses of the number of direct and water-mediated H-bonding of M159 E4A and M159 E8V during the production runs. We present the total number of direct H-bonds between peptide sidechains (self and non-self), and water-mediated bridges between sidechains with up to three H-bonded water molecules per bridge. All H-bonds sampled at least once, regardless of the average H-bond occupancies, are included in these time series. The origin of time is the start of the production runs without any constraints. Black and blue profiles represent time courses computing from the main and repeat simulations, respectively. (A, B) Time series computed for macrolittin M159 E4A (panel A) and M159 E8V (panel B).

M159 E4A Repeat Simulation

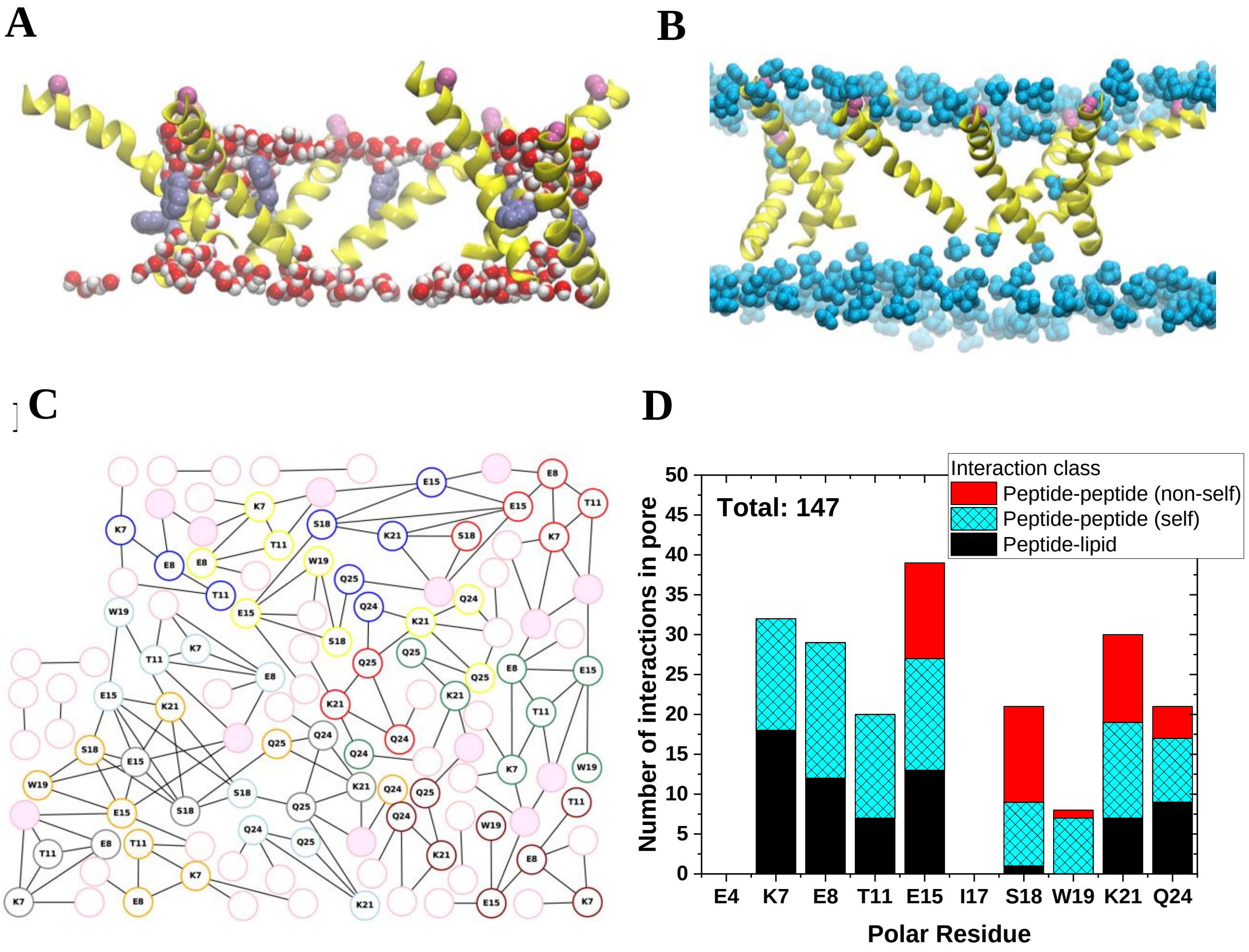

Figure S11. Results of repeat simulation of macrolittin M159 E4A. (A) Cut-away views showing water molecules interacting in the M70 pore. Peptides are shown as yellow ribbons, and water molecules are shown as van der Waals spheres with oxygen and H atoms colored red and white, respectively. The W19 sidechain of each peptide is shown as van der Waals spheres; for clarity, H atoms of the W19 sidechains are not shown. (B) Cut-away view illustrating lipid phosphate groups. (C,D) H-bond graph computed from the last ~200ns of the repeat simulation of macrolittin M159 E4A. For clarity, we include only H-bonds with a minimum occupancy of 30%. Detailed information on the average occupancy and average number of water molecules for each edge of the graph is presented in Figure S14. (D) Summary of the number of H-bonds counted based on the H-bond graph presented in panel C.

M159 E8V Repeat Simulation

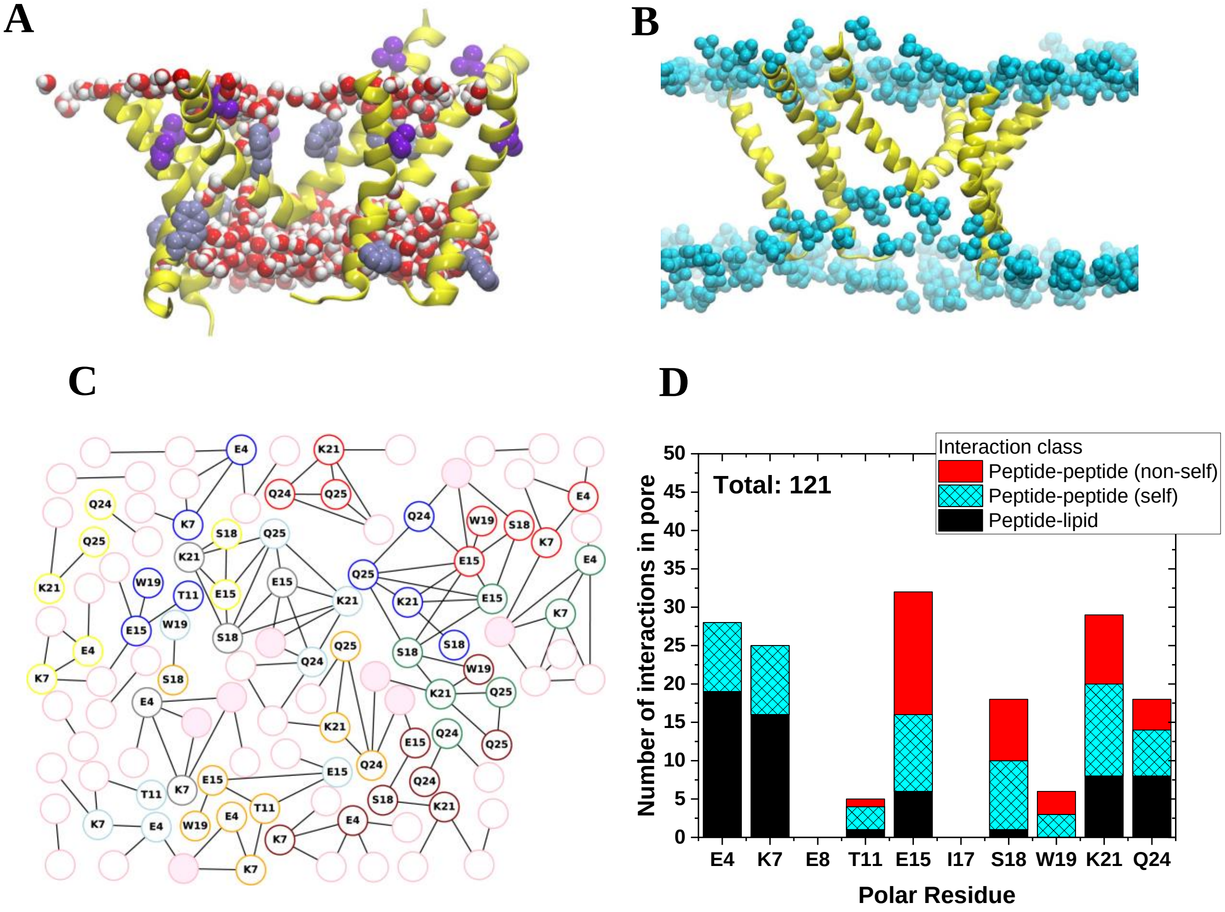

Figure S12. Results of repeat simulation of macrolittin M159 E8V. (A) Cut-away views showing water molecules interacting in the M70 pore. Peptides are shown as yellow ribbons, and water molecules are shown as van der Waals spheres with oxygen and H atoms colored red and white, respectively. The W19 sidechain of each peptide is shown as van der Waals spheres; for clarity, H atoms of the W19 sidechains are not shown. (B) Cut-away view illustrating lipid phosphate groups. (C,D) H-bond graph computed from the last ~200ns of the repeat simulation of macrolittin M159 E8V. For clarity, we include only H-bonds with a minimum occupancy of 30%. Detailed information on the average occupancy and average number of water molecules for each edge of the graph is presented in Figure S16. (D) Summary of the number of H-bonds counted based on the H-bond graph presented in panel C.

M159 E4A Main Simulation

Average waters per bridge

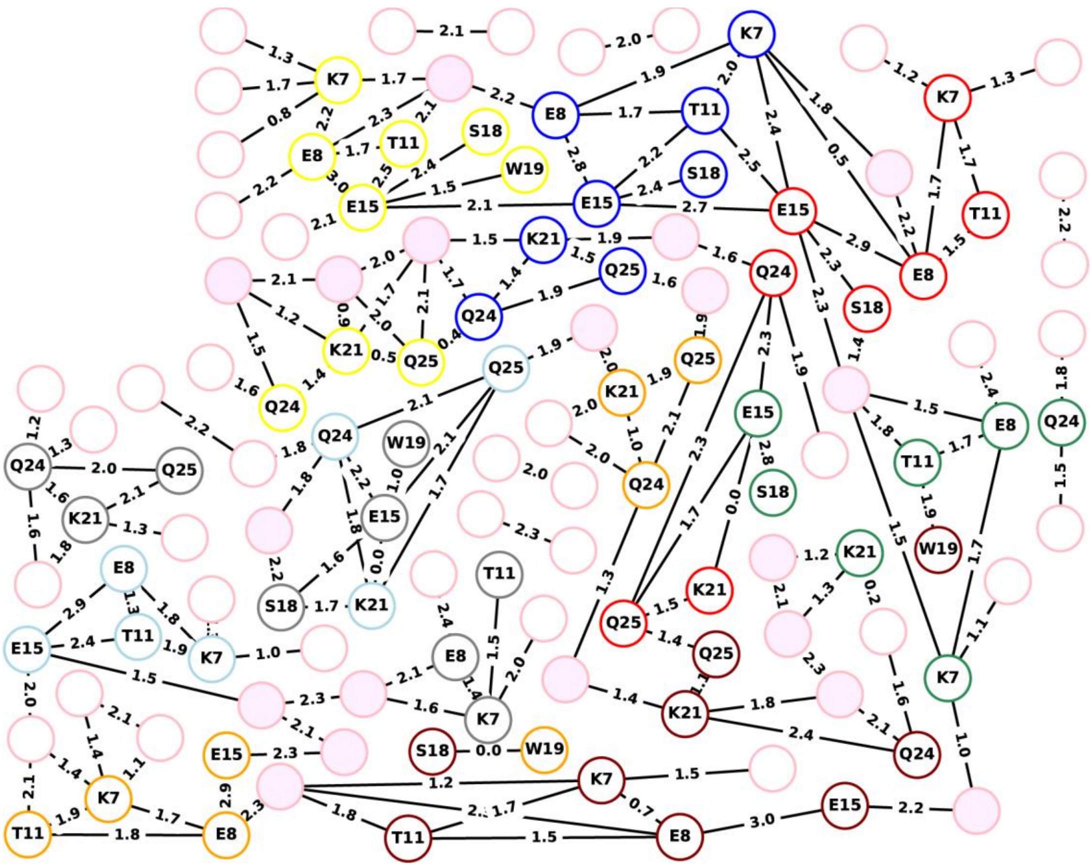

Average % occupancy

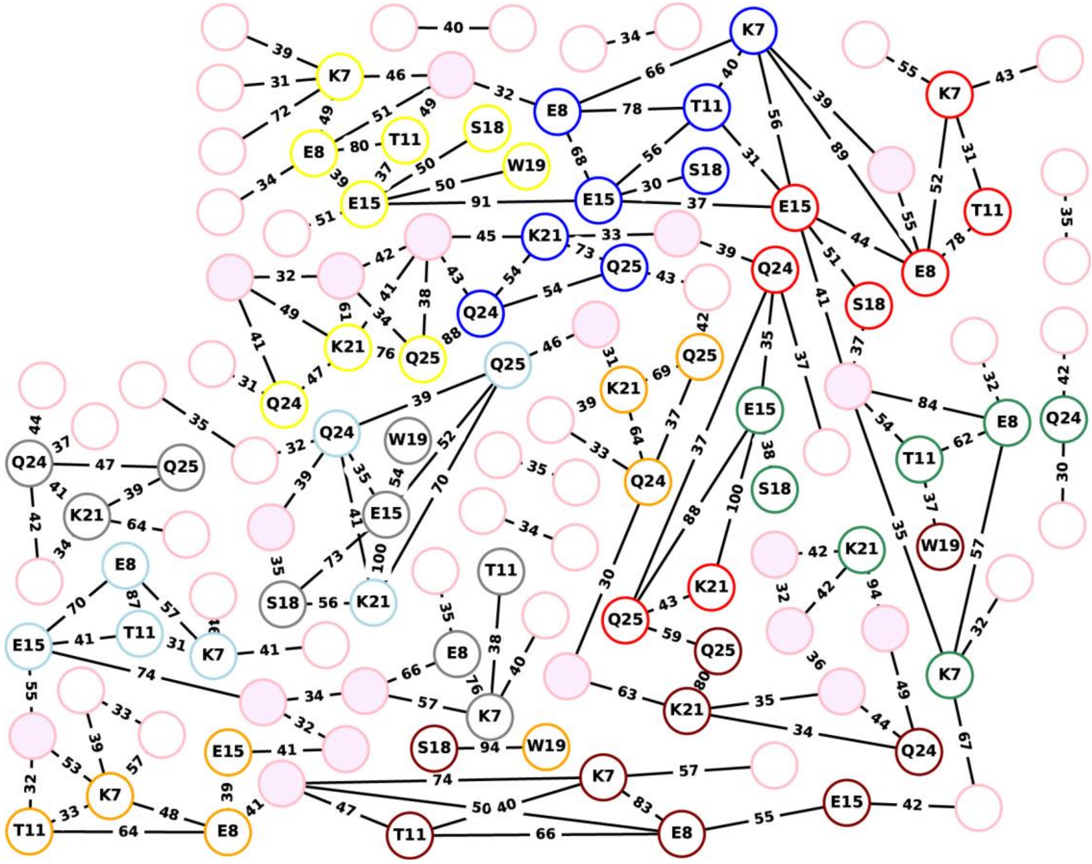

Figure S13. Results of the main simulation of macrolittin M159 E4A. These images display the average number of water molecules in the H-bonded bridges (top) and the average % occupancy of each direct or water bridged H-bond (bottom). Values were computed from the last ~200ns of the main simulation of macrolittin M159 E4A. For clarity, we include only H-bonds with a minimum occupancy of 30%.

M159 E4A Repeat Simulation

Average waters per bridge

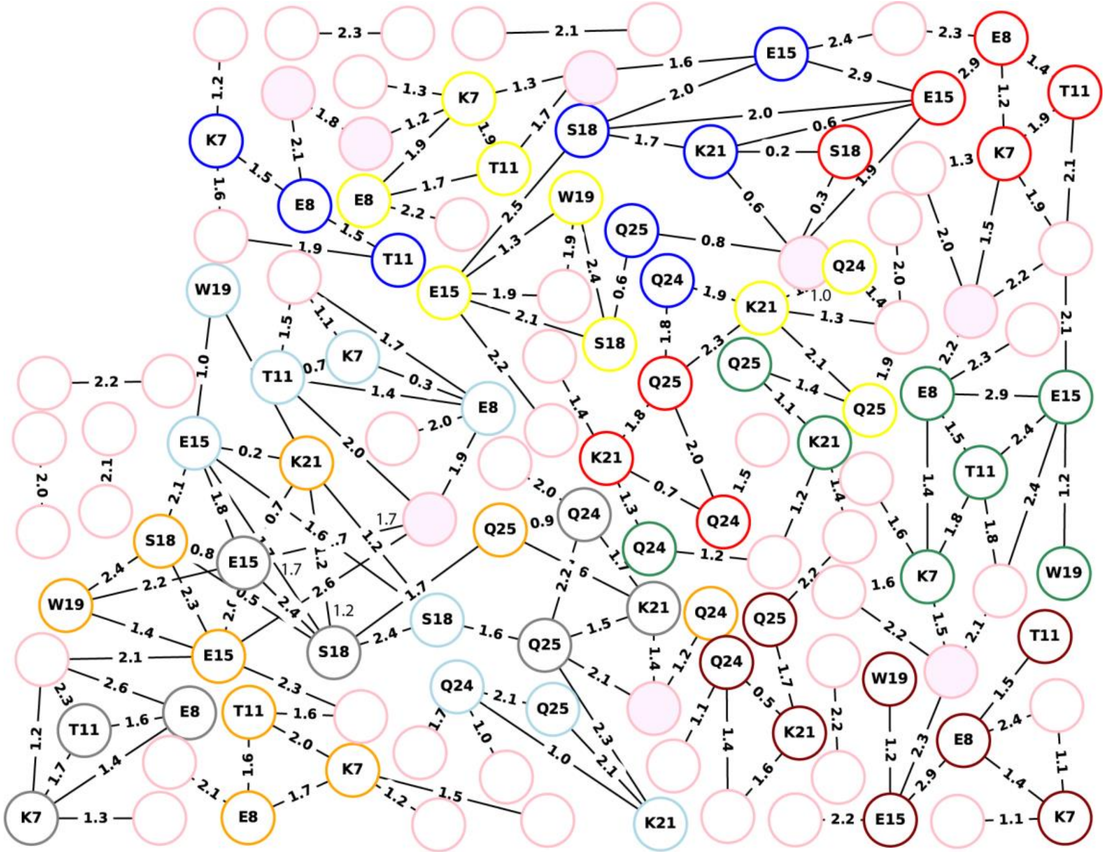

Average % occupancy

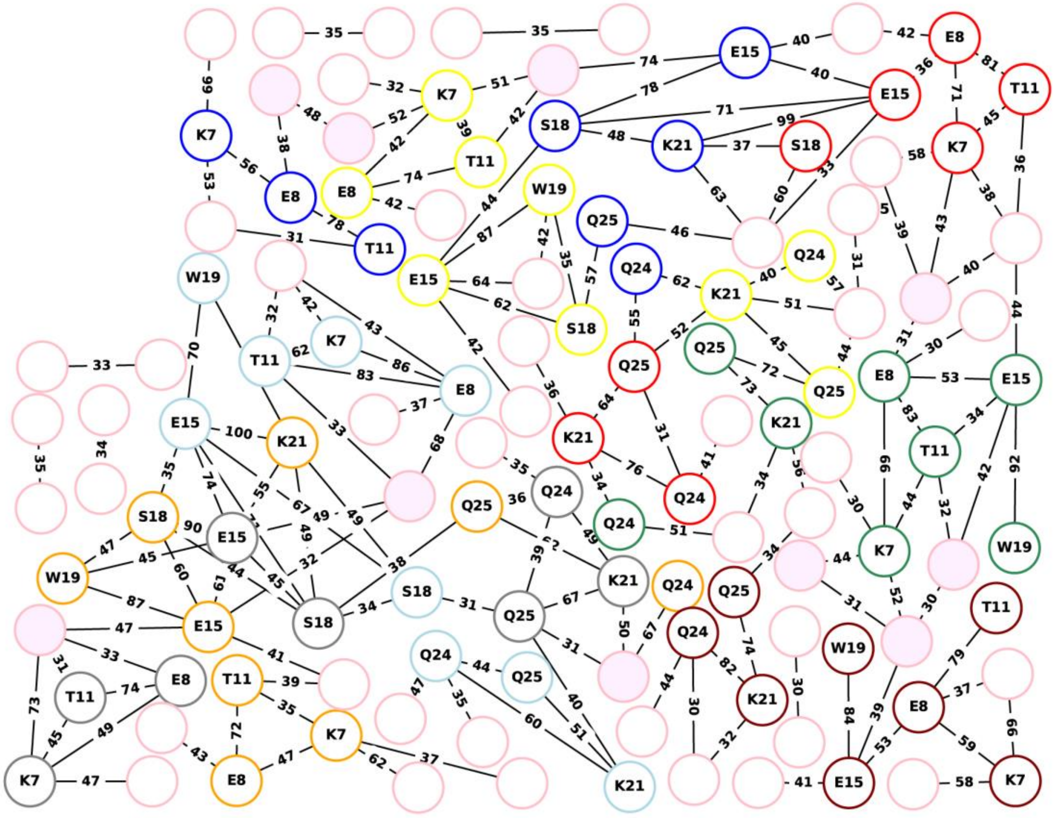

Figure S14. Results of the repeat simulation of macrolittin M159 E4A. These images display the average number of water molecules in the H-bonded bridges (top) and the average % occupancy of each direct or water bridged H-bond (bottom). Values were computed from the last ~200ns of the repeat simulation of macrolittin M159 E4A. For clarity, we include only H-bonds with a minimum occupancy of 30%.

M159 E8V Main Simulation

Average waters per bridge

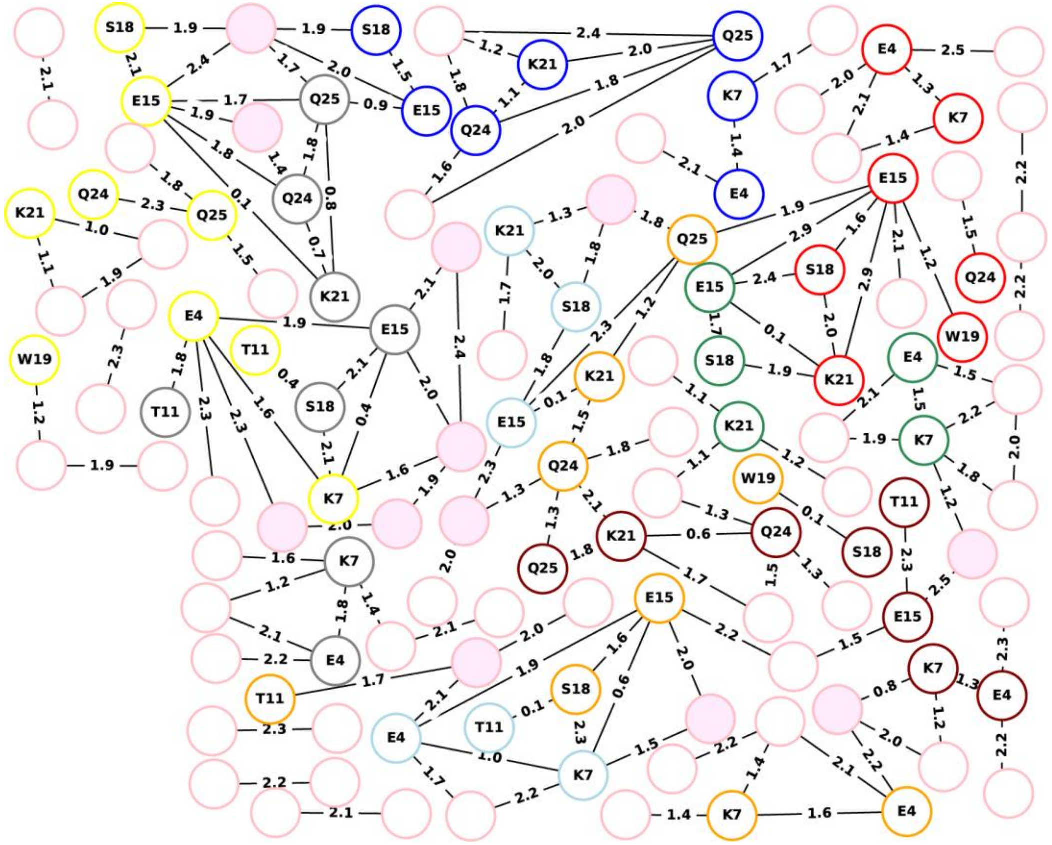

Average % occupancy

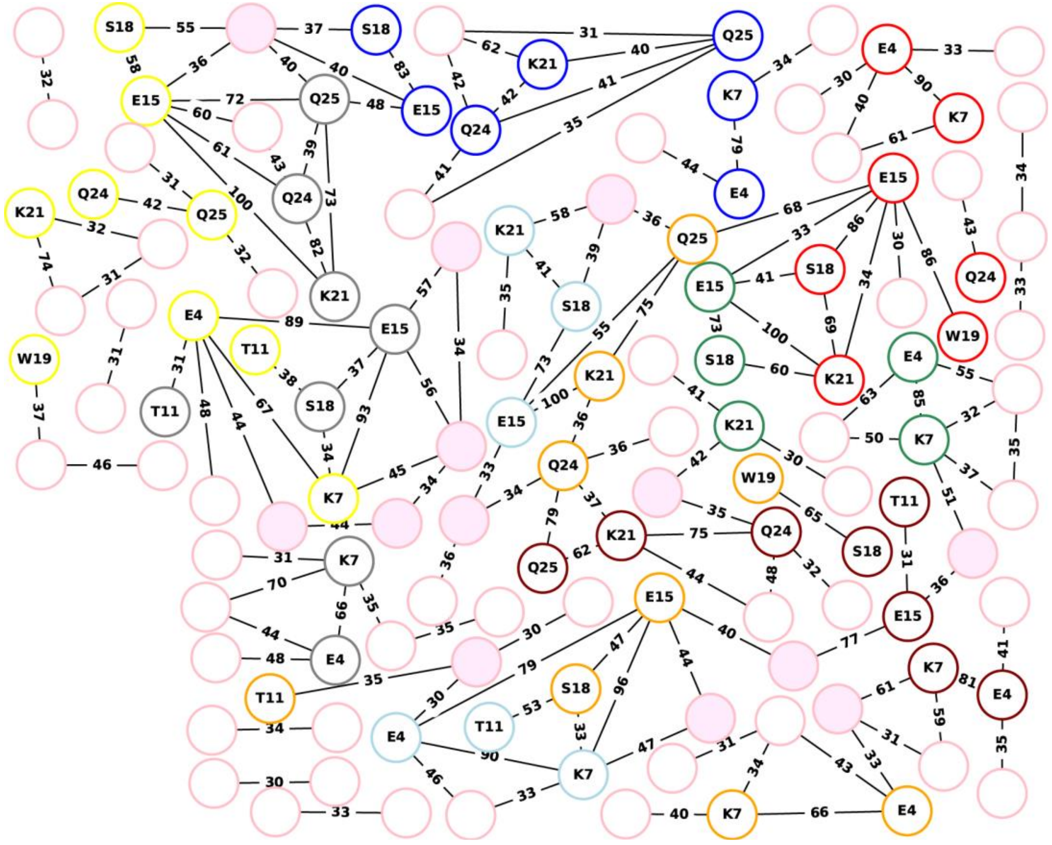

Figure S15. Results of the main simulation of macrolittin M159 E8V. These images display the average number of water molecules in the H-bonded bridges (top) and the average % occupancy of each direct or water bridged H-bond (bottom). Values were computed from the last ~200ns of the main simulation of macrolittin M159 E8V. For clarity, we include only H-bonds with a minimum occupancy of 30%.

M159 E8V Repeat Simulation

Average waters per bridge

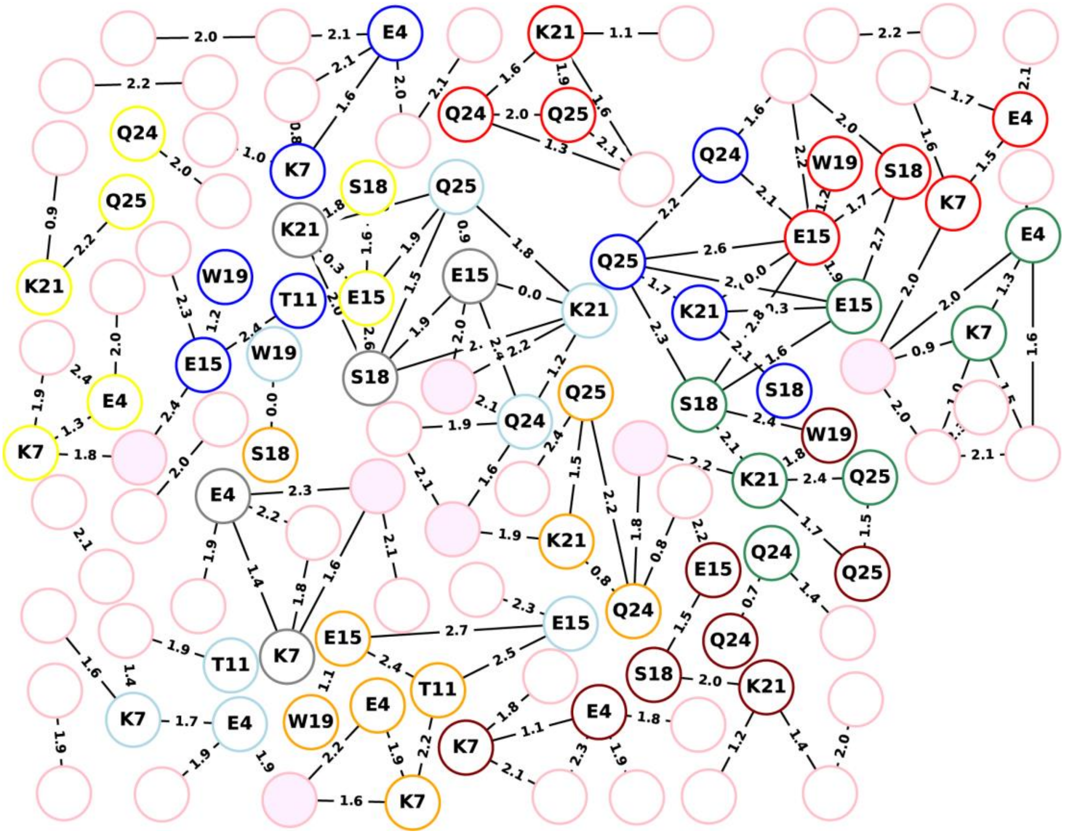

Average % occupancy

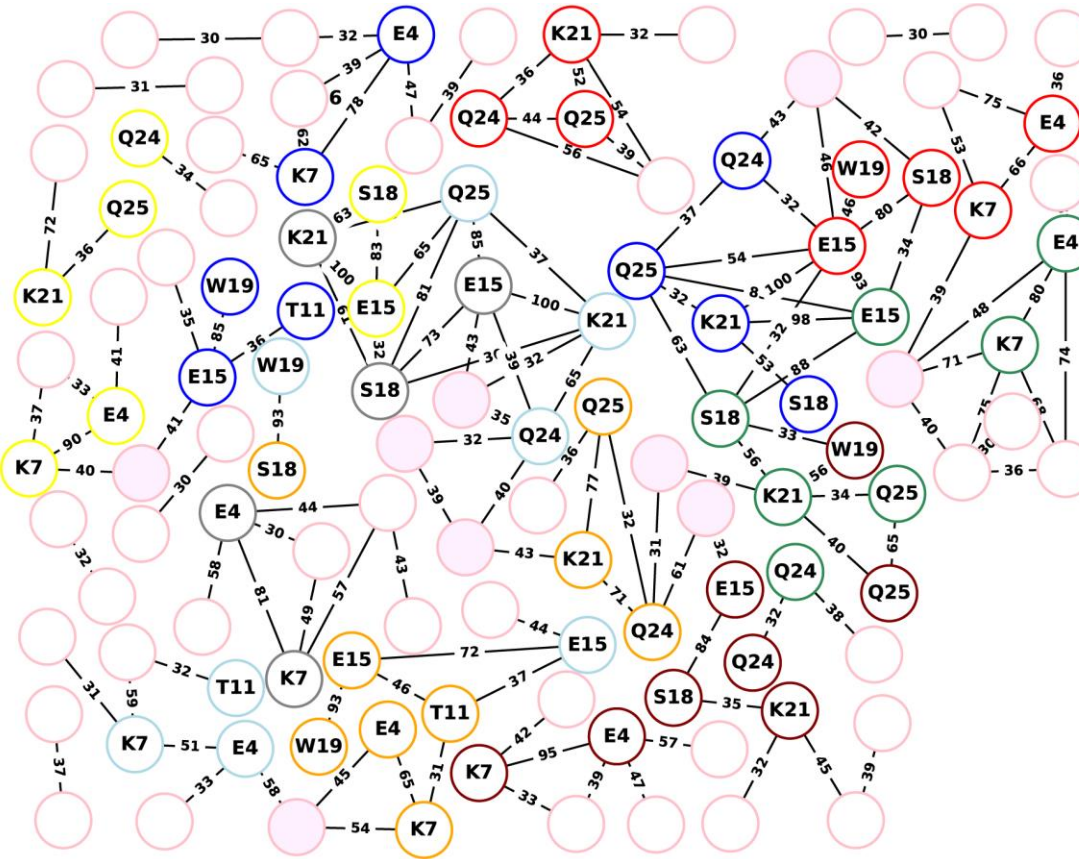

Figure S16 Results of the repeat simulation of macrolittin M159 E8V. These images display the average number of water molecules in the H-bonded bridges (top) and the average % occupancy of each direct or water bridged H-bond (bottom). Values were computed from the last ~200ns of the repeat simulation of macrolittin M159 E8V. For clarity, we include only H-bonds with a minimum occupancy of 30%.

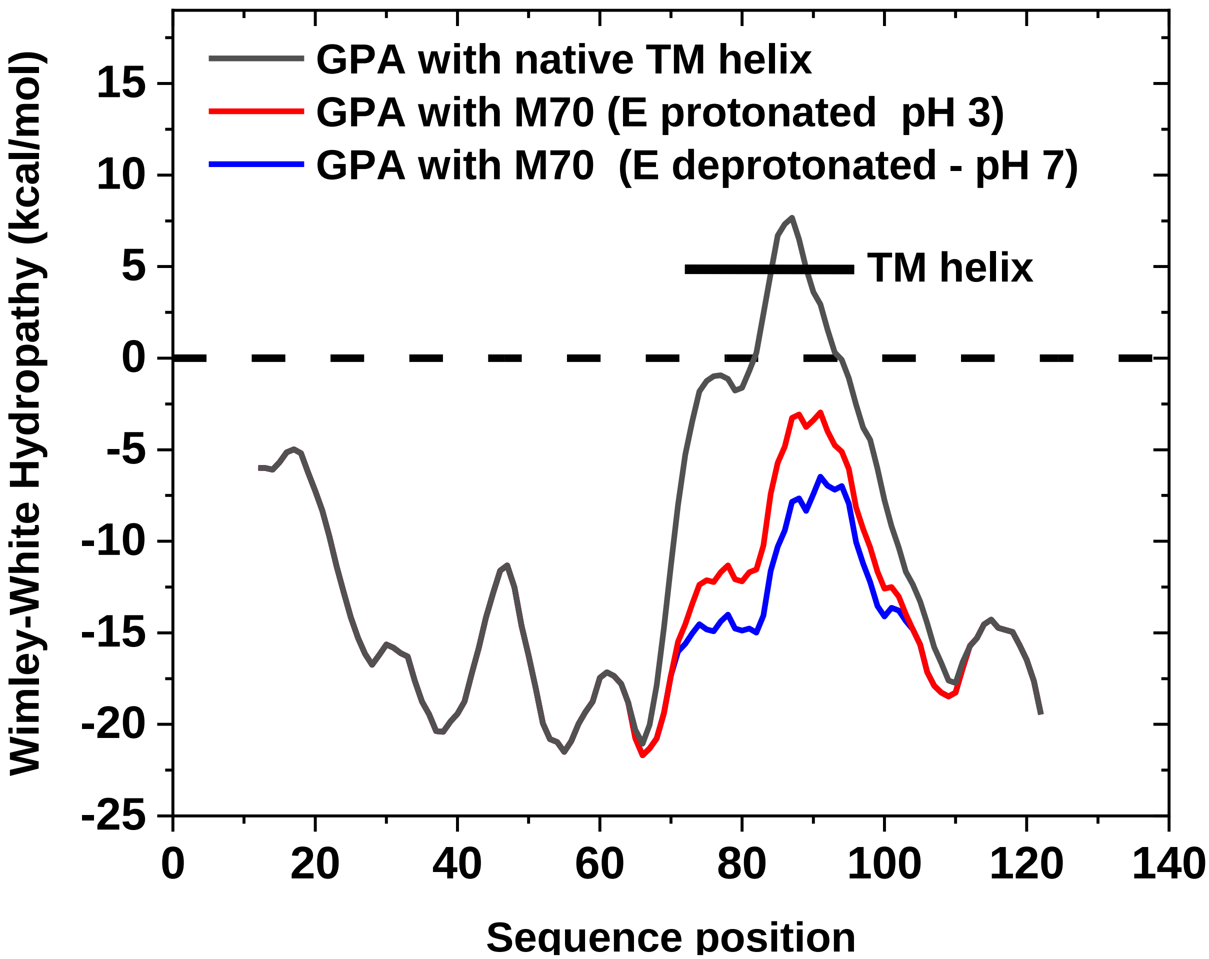

Figure S17. Sliding window hydropathy plot for the classical single span membrane protein glycophorin A is shown in black. The TM helix of GPA has positive hydrophobicity. In red, the TM helix of glycophorin A is replaced by the sequence of macrolittin M70, with the glutamates protonated, equivalent to pH << 4. In blue, the TM helix of glycophorin A is replaced by the sequence of macrolittin M70, with the glutamates deprotonated, equivalent to neutral pH. In both cases the sequence of M70 is not hydrophobic enough to be identified as a transmembrane helix.
